# Two passive mechanical conditions modulate power generation by the outer hair cells

**DOI:** 10.1371/journal.pcbi.1005701

**Published:** 2017-09-07

**Authors:** Yanju Liu, Sheryl M. Gracewski, Jong-Hoon Nam

**Affiliations:** 1 Department of Mechanical Engineering, University of Rochester, Rochester, New York, United States of America; 2 Department of Biomedical Engineering, University of Rochester, Rochester, New York, United States of America; Georgia Institute of Technology, UNITED STATES

## Abstract

In the mammalian cochlea, small vibrations of the sensory epithelium are amplified due to active electro-mechanical feedback of the outer hair cells. The level of amplification is greater in the base than in the apex of the cochlea. Theoretical studies have used longitudinally varying active feedback properties to reproduce the location-dependent amplification. The active feedback force has been considered to be proportional to the basilar membrane displacement or velocity. An underlying assumption was that organ of Corti mechanics are governed by rigid body kinematics. However, recent progress in vibration measurement techniques reveals that organ of Corti mechanics are too complicated to be fully represented with rigid body kinematics. In this study, two components of the active feedback are considered explicitly—organ of Corti mechanics, and outer hair cell electro-mechanics. Physiological properties for the outer hair cells were incorporated, such as the active force gain, mechano-transduction properties, and membrane RC time constant. Instead of a kinematical model, a fully deformable 3D finite element model was used. We show that the organ of Corti mechanics dictate the longitudinal trend of cochlear amplification. Specifically, our results suggest that two mechanical conditions are responsible for location-dependent cochlear amplification. First, the phase of the outer hair cell’s somatic force with respect to its elongation rate varies along the cochlear length. Second, the local stiffness of the organ of Corti complex felt by individual outer hair cells varies along the cochlear length. We describe how these two mechanical conditions result in greater amplification toward the base of the cochlea.

## Author summary

The mammalian cochlea encodes sound pressure levels over six orders of magnitude. This wide dynamic range is achieved by amplifying weak sounds. The outer hair cells, one of two types of receptor cells in the cochlea, are known as the cellular actuators that provide power for the amplification. It is well known that high frequency sounds encoded in the basal turn of the cochlea are amplified more than low frequency sounds encoded in the apical turn of the cochlea. This difference in amplification has been ascribed to a difference in electrophysiological properties, such as the membrane capacitance and conductance of the outer hair cells at different locations. Whether the outer hair cells have a sufficient range of electrophysiological properties to explain the location dependent amplification has long been a topic of scientific debate. In this study, we present an alternative explanation for how the low and high frequency sounds are amplified differently. Using a detailed computational model of the cochlear epithelium (the organ of Corti), we demonstrate that the micro-mechanics of the organ of Corti can explain the variation of amplification with longitudinal location in the cochlea.

## Introduction

The mammalian cochlea encodes sounds with pressure levels ranging over six orders of magnitude into neural signals. This wide dynamic range of the cochlea is achieved by the amplification of low amplitude sounds. The outer hair cells have been identified as the mechanical actuators that generate the forces needed for cochlear amplification [1]. Cochlear amplification is dependent on location along the cochlear length. For example, according to measurements of the chinchilla cochlea, the amplification factor of basilar membrane (BM) vibrations was about 40 dB in basal locations while it was 15 dB in apical locations [2–4].

Theoretical studies have reproduced location-dependent cochlear amplification by adopting tonotopic electrophysiological properties, such as the active feedback gain of the outer hair cells [5, 6], or the mechano-transduction properties of the outer hair cell stereocilia [7, 8]. These studies are based on experimental reports concerning the tonotopy of the outer hair cells’ electrophysiological properties [e.g., 9, 10–12]. On the other hand, recent experimental observations suggest that organ of Corti mechanics may play a role in cochlear amplification. For example, organ of Corti micro-structures vibrate either in phase or out of phase depending on stimulation level and frequency [13–16]. These observations challenge a long-standing framework for modeling the organ of Corti mechanics—rigid body kinematics, introduced by ter Kuile [17]. A fully deformable organ of Corti may have implications for cochlear amplification.

Micro-mechanical aspects of cochlear power amplification were investigated in our previous study, using a computational model of the cochlea [18]. The model features detailed organ of Corti mechanics analyzed using a 3-D finite element method, and up-to-date outer hair cell physiology. In that previous work [18], it was shown that the stiffness of the organ of Corti complex (OCC) felt by the outer hair cells remains comparable to the outer hair cell stiffness, independent of location. An intriguing observation was that even though the same active force gain was used for all outer hair cells, the model reproduced greater amplification toward the base. However, the specific model aspects responsible for the location-dependence were not identified in that paper. In this study, by analyzing power generation in individual hair cells, by observing different micro-mechanical transfer functions of the organ of Corti, and through a series of parametric studies, we identify passive mechanical aspects that are responsible for the location-dependent amplification.

## Results

In the following, three longitudinal locations: *x* = 2, 6, and 10 mm are referred to as the base, middle, and apex of the gerbil cochlea, respectively. By active or passive responses, we mean the responses with or without the outer hair cell active force, respectively. For all simulations, the stapes velocity amplitude is 1 nm/ms. The ‘stapes velocity’ is defined as the kinematic boundary condition along the edge at *x* = 0 and 0 < *y* < *H*, where *H* is the height of the top and the bottom fluid chamber. In this study, *H* = 0.3 mm. Because this study uses a linearized frequency-domain model, nonlinear level-dependent effects were not simulated. Electromechanical parameters at the state of equilibrium were chosen so that our ‘active cochlea’ is comparable to sensitive cochlear under small sound stimuli, while the passive responses are comparable to postmortem responses.

### Fluid dynamical, micro-mechanical and electrical responses at different locations

**Fig 1:**
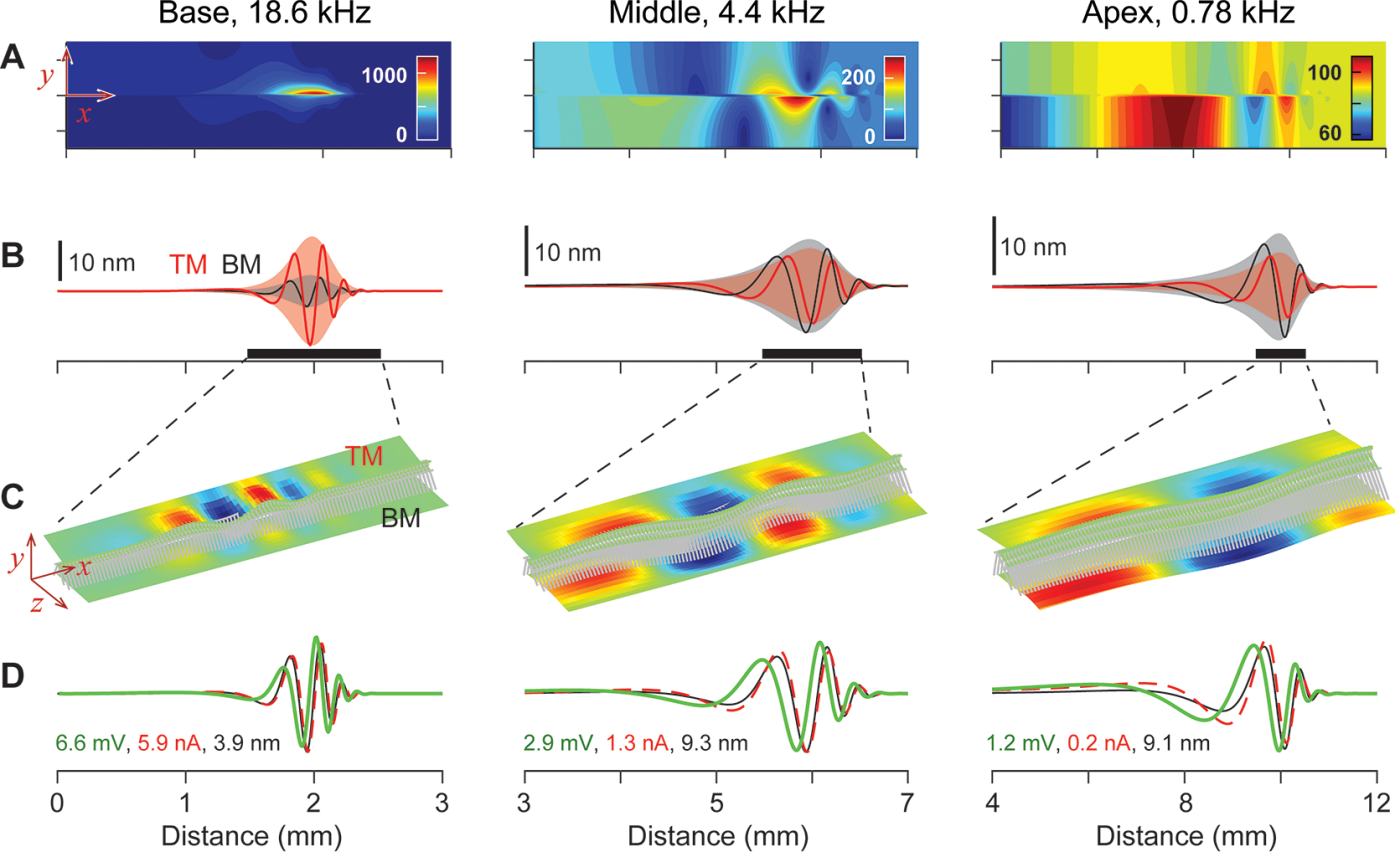
Fluid dynamical, micro-structural and electrical responses of the cochlea: Active case. The three columns represent the responses at the base, middle and apex respectively. When the stapes is vibrated at 18.6, 4.4 and 0.78 kHz, the responses peak at x = 2, 6 and 10 mm, respectively. The stapes velocity amplitude is 1 nm/ms. (**A**) Cochlear fluid pressure amplitude referenced to the pressure at the round window. The unit for the pressure scale is mPa. (**B**) Spatial pattern of the BM (basilar membrane) and the TM (tectorial membrane) vibration at the same moment of time. (**C**) Vibration patterns of the 3-D finite element model of the OCC. A 1-mm section around the peak (thick bars above the x-axes in (B)) is shown. The color contours of the top (TM) and the bottom (BM) structures indicate transverse displacement amplitude at a moment of time. Red and blue colors represent the displacement toward the scala vestibule and toward the scala tympani, respectively. (**D**) Normalized amplitudes of membrane potential (green) and mechano-transduction current (red) of the outer hair cells plotted together with the BM displacement (black). The numbers correspond to the peak amplitudes of receptor potential (in mV), transduction current (in nA) and BM displacement (in nm).

**Fig 2:**
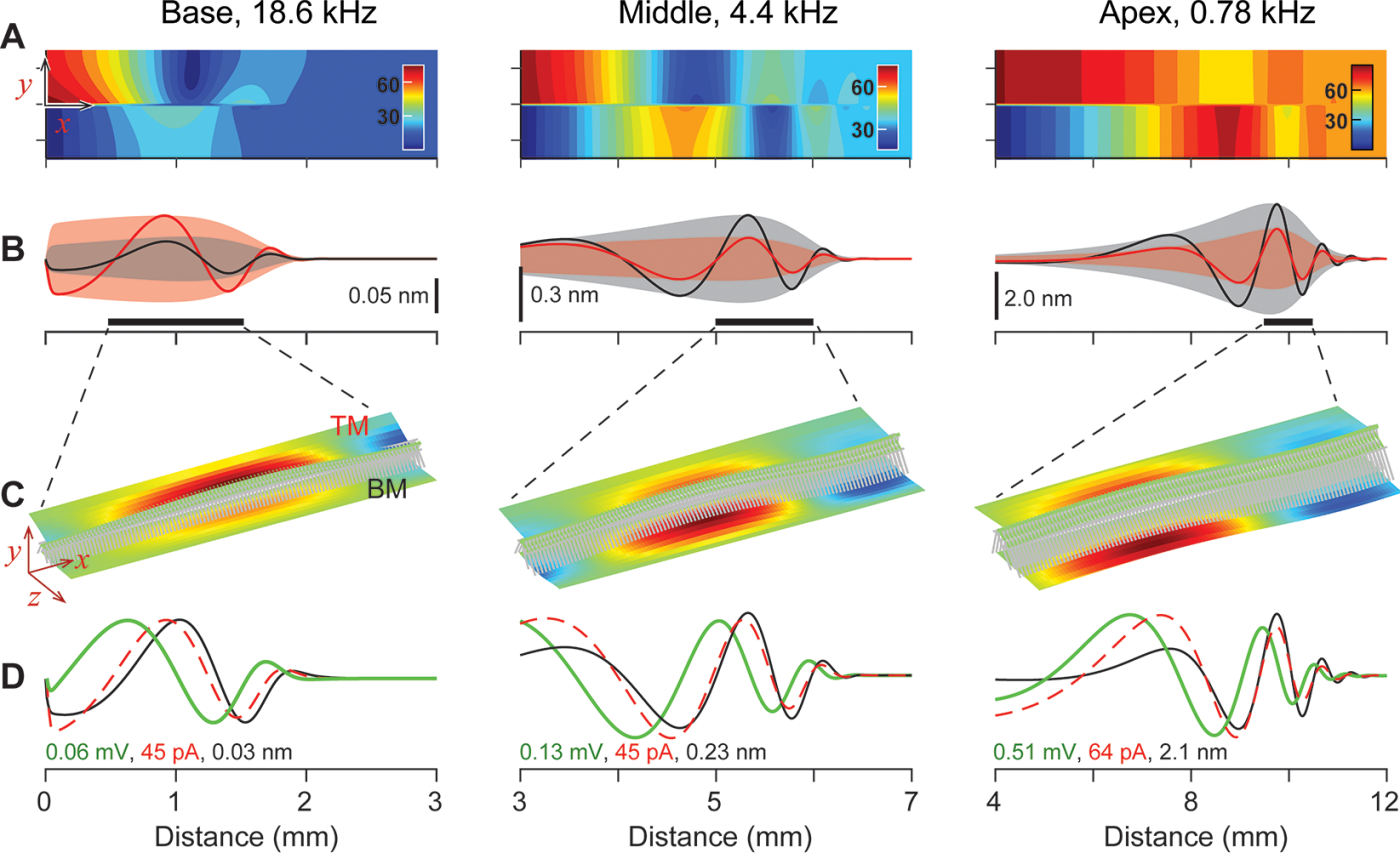
Fluid dynamical, micro-structural and electrical responses of the cochlea: Passive case. The same conditions as Fig 1, except that the results were obtained without the active force of the outer hair cells. (**A**) Cochlear fluid pressure amplitude. (**B**) Spatial pattern of the BM (basilar membrane) and the TM (tectorial membrane) vibration. (**C**) Vibration patterns of the 3-D finite element model of the OCC. (**D**) Normalized amplitudes of membrane potential (green) and mechano-transduction current (red) of the outer hair cells plotted together with the BM displacement (black).

Two sets of fluid dynamical, structural and electro-physiological responses are presented in Fig 1 and Fig 2 that represent the active and the passive responses, respectively. When the stimulating frequency was 18.6, 4.4 and 0.78 kHz, the BM vibrations of the active cochlea peaked at *x* = 2, 6 and 10 mm, respectively (Fig 1). For the same stimulating frequencies, the peak responding location shifted toward the base when passive (*x* = 1.2, 5.4, 9.7 mm, Fig 2). This shift of peak responding location due to the outer hair cell active feedback is consistent with experimental observations [e.g., 19], and other model studies [e.g., 20, 21].

The pressure amplitude plots show one or two peaks and notches as the pressure propagates from the oval window toward the apex. This is similar to the pressure peaks/notches observed experimentally by Kale and Olson [22]. As was discussed in their work, the pressure peaks/notches were generated by the interference between fast and slow pressure components. For example, the slow (differential pressure across the OCC) component showed no pressure notch. When the amplification is large (Fig 1A), the local pressure near the peak responding location was large enough to mask the pressure pattern created by the oval window motion. Despite changes in the outer hair cell active force, the pressure at the stapes remains relatively constant at about 80 mPa for a 1 nm/ms stapes velocity amplitude. After considering the stapes footplate area of 0.8 mm^2^ [23], our simulated result corresponds to a cochlear input impedance of 100 GPa·s/m^3^. This value is comparable to measured values ranging between 50 and 300 GPa·s/m^3^ [24–26].

Explicit computation of the interactions between the outer hair cells and OCC fine structures is both an opportunity and a challenge of our continuum mechanics-based approach. Any poorly determined parameters can affect the result, and there are a large set of model parameters. However, as more OCC mechanical data accumulate, well defined parameters can serve as rigorous constraints on the model. For example, the geometrical information of the OCC is well known, but underused. The elastic moduli or stiffness of different OCC structures have been measured as summarized in [27]. This study takes advantage of such existing data. As a result, the vibration amplitude ratios and phase relationships between the micro mechanical structures vary depending on location, simulating frequency, and active force feedback.

The resulting micro-mechanical responses compare well with experimental observations. For example, the vibration pattern (the relative motion between different structural components) changes depending on the outer hair cell’s active feedback: when active, the TM (tectorial membrane) vibrations lead the BM vibrations by 15 to 60 degrees (Fig 1B), but they vibrate in phase when passive (Fig 2B). This dependence of vibration patterns on the outer hair cell motility has been observed experimentally in the gerbil cochlea [13]. Our model predicts that the micro-mechanical response characteristics are location-dependent: at middle to apical locations, the TM vibrates less than the BM, but the opposite is true in the basal turn (*x* < 4 mm). The relative motion between the BM and other OCC structures, caused by the outer hair cell’s active feedback, indicates that the top and the bottom of the OCC are effectively decoupled. In the field of vibration measurement, the vibration patterns due to internal forces are referred to as the operational deflection shapes. This decoupling due to the outer hair cell action persists along the entire cochlear length [28].

Up-to-date physiological properties of outer hair cell mechano-transduction and electromotility are incorporated into our model to predict the electro-mechanical feedback of the cells to acoustic stimulations. The amplitudes of the mechano-transduction current and receptor potential are presented in Fig 1D, and Fig 2D. Independent of location or outer hair cell feedback force, mechano-transduction current is nearly in phase with the BM displacement.

Using these first-hand results (fluid pressure, vibration amplitudes of micro-structures, and electrical responses of the outer hair cells), we analyzed how the outer hair cells’ power generation is modulated by the organ of Corti mechanics.

### Location-dependent amplification

**Fig 3:**
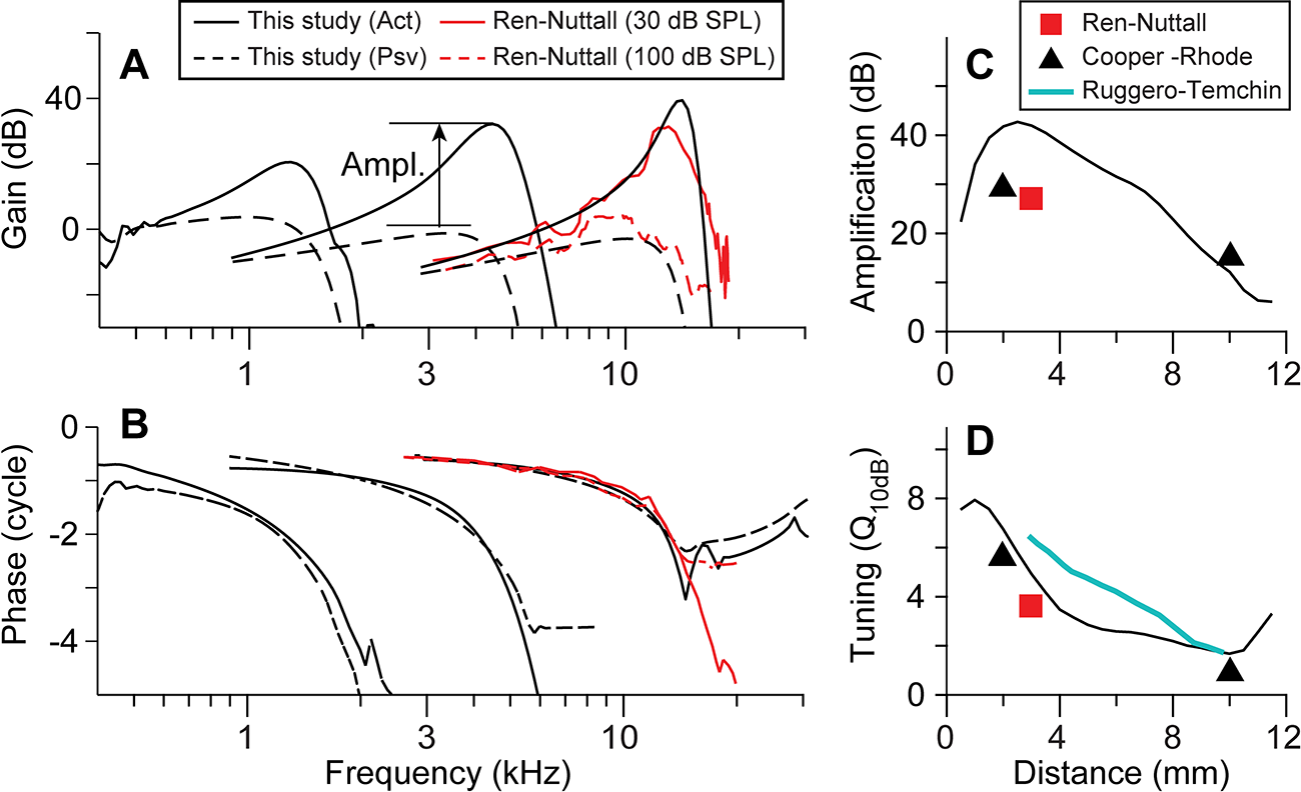
Location-dependent amplification and tuning. Simulated responses to pure tone stimulations were plotted together with experimental results. (**A**) The displacement gain of the BM with respect to the stapes. The gain at four different locations (*x* = 9, 6, and 3 mm) versus stimulating frequency. The amplification is defined as the gain difference between the active and the passive peak responses. The experimental results were shifted downward by 17 dB for comparison. (**B**) The phase of the BM displacement with respect to the stapes. (**C**) Amplification factor versus longitudinal position. (**D**) Tuning quality versus longitudinal position. Experimental data: Ren & Nuttall (2001), Cooper & Rhode (1997), Ruggero & Temchin (2005). Cooper-Rhode data from Chinchilla are compared with similar best-frequency-locations of the gerbil cochlea (17 and 0.5 kHz at 2.2 and 11 mm, respectively).

Model responses at three different locations (*x* = 9, 6, and 3 mm) are presented together with experimental results in Fig 3. There are measurements of the gerbil cochlear vibrations at different locations [e.g., 19, 29]. Cooper and Rhode’s experiment with the chinchilla cochlea [4] is also pertinent to this study, because the mechanical amplification measurements from different cochlear locations were reported in a single paper.

Our simulated results for two key quantitative measures that represent cochlear performance are in reasonable agreement with experimental observations [4, 19, 29]. First, the BM vibrations are amplified by 30–50 dB in high-frequency locations (*x* < 4 mm), and 10–20 dB in low-frequency locations (*x* > 8 mm). Second, the tuning quality represented by Q_10dB_ are > 3 in the base, and ~1 in the apex. While gain and phase curve shapes are in reasonable agreement with the experiment, there are differences between our model response and experimental results in absolute values. As compared to Ren and Nuttall’s measurements [19], the gain was lower by 17 dB, and the phase was different by a half cycle. The difference in gain may be ascribed to different reference input conditions: our kinematic boundary condition at *x* = 0 and 0 < *y* < *H*, does not exactly represent the stapes excitation. The half a cycle phase difference between simulation and experimental results could be due to different definitions of positive stapes displacement. In this study, the stapes velocity is positive when moving into the cochlea.

The phase of BM vibration versus stimulating frequency has been extensively measured and analyzed because it characterizes the cochlear traveling waves [e.g., 30, 31]. Three key characteristics are reproduced by our simulations in Fig 3B: First, there exist more than two cycles of total phase accumulation as the frequency increases. Second, the phase accumulation at the peak responding location is between 1 and 3 cycles. Finally, the slope of the phase versus frequency curve is similar for the active and passive cases.

The amplification and tuning quality of the cochlea decrease toward the apex (Fig 3C and 3D). Because the amplification is the primary consequence of the outer hair cell’s active feedback, the location-dependent amplification implies that the outer hair cells provide more power in the base. One possible approach to model this location-dependent amplification is to assume that the outer hair cells in the basal cochlea have greater active force gain than those in the apex. Alternatively, the passive mechanics of the OCC may be responsible for the location-dependent amplification. Note that our model adopted a constant force gain (*g*_*OHC*_ of 0.1 nN, active force per mV membrane potential change after [11]) independent of location. We investigated which location-dependent properties could be responsible for the greater amplification in the base.

### Power generated by individual outer hair cells

**Fig 4:**
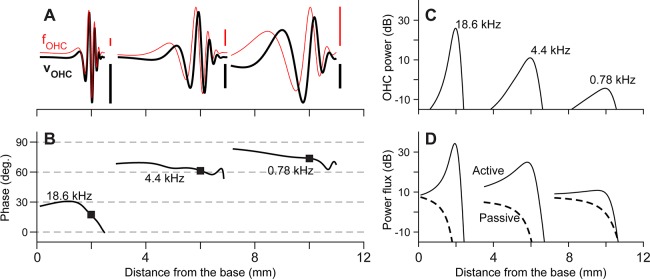
Power generated by outer hair cells. At three different stimulating frequencies (18.6, 4.4 and 0.78 kHz), the outer hair cell active force (f_OHC_) and the rate of outer hair cell elongation (v_OHC_) were computed. The product of these two properties defines the power generated by individual outer hair cells. (**A**) The f_OHC_ (thin curves) and v_OHC_ (thick curves) at a moment of time versus distance from the base for three stimulating frequencies. The upper vertical scale bars indicate 0.1 nN. The lower scale bars indicate 1, 0.1, 0.01 mm/s from the left to the right. (**B**) Phase of v_OHC_ with respect to f_OHC_. The square markers indicate the peak locations. (**C**) Power generated by individual hair cells at different locations. The unit is dB re 1 fW (10^−15^ Watt). (**D**) Power flux at different locations. The unit is dB re 1 fW (10^−15^ Watt).

To investigate the origin of the location-dependent amplification, power generation by individual outer hair cells was analyzed (Fig 4). The power provided by an outer hair cell to its external system is defined as the product of the active force generated by the cell (*f*_*OHC*_) and the rate of cell’s length change (*v*_*OHC*_, see Eq (6) in Methods). In Fig 4A, *f*_*OHC*_ and *v*_*OHC*_ at a moment of time are presented for high, mid and low frequency stimulations (18.6, 4.4, and 0.78 kHz). For a stapes velocity of 1 nm/ms, the peak values of *f*_*OHC*_ and *v*_*OHC*_ are 0.65 nN and 1.2 mm/s at 18.6 kHz, and 0.11 nN and 21 μm/s at 0.78 kHz. This trend of decreasing force and velocity toward lower stimulating frequency is consistent with decreased amplification toward the apex.

The timing (phase) of outer hair cell force generation also contributes to the location-dependent amplification in the cochlea. The phase of *v*_*OHC*_ with respect to *f*_*OHC*_ depends on stimulating frequency (Fig 4B). At the peak-responding location, *f*_*OHC*_ lags *v*_*OHC*_ by 17, 61 and 83 degrees for stimulating frequencies of 18.6, 4.4 and 0.78 kHz, respectively. For given *f*_*OHC*_ and *v*_*OHC*_ amplitudes, the power generation by an outer hair cell is greatest when *f*_*OHC*_ is in phase with *v*_*OHC*_, and zero when they are 90 degrees out of phase. In other words, the *f*_*OHC*_-*v*_*OHC*_ phase causes the outer hair cells in the base to be 8 times more efficient in generating power than those in the apex (cos(17°)/cos(83°) ≈ 8). The power generation per cycle of individual outer hair cells (computed from Eq (6) in Methods) is shown in Fig 4C. For a stapes vibration amplitude of 1 nm/ms, an individual outer hair cell at the peak responding location generates 377, 12, and 0.36 fW for the three stimulating frequencies of 18.6, 4.4 and 0.78 kHz, respectively. The apical value is comparable to values reported by Wang *et al*. [32], but the basal value is about two to three orders of magnitude greater. This discrepancy could be due to the difference in model species (Wang *et al*. modeled the mouse cochlea). As Wang *et al*. discussed, small difference in vibration amplitude can result in different estimations of outer hair cell power generation (*i*.*e*., the gerbil cochlea may vibrate greater than the mouse cochlea). Alternatively, the difference may be ascribed to difference in model assumptions such as dissipating mechanisms. While we lumped the effect of power loss due to the viscous fluid with the viscous damping of the OCC structures, Wang and her colleagues incorporated the fluid viscosity explicitly and considered the damping within the OCC negligible. Further investigation of the OCC mechanical impedance may be required to better understand the difference.

The power flux (computed using Eq (1) in Methods) represents how much energy is transferred by the scala fluid along the cochlear length. In the passive system, the power is provided through the stapes. As a result, the longitudinally transmitted power is dissipated as the traveling waves propagate. That is, the power flux decreases monotonically toward the apex when passive (dashed curves, Fig 4D). However, for the active cochlea (comparable to the case of weak sound stimulation to healthy ears), the power flux pattern is non-monotonic—the power flux increases until it culminates at the best responding location (solid curves, Fig 4D). A similar trend of power flux was shown in other theoretical studies [32, 33].

### Passive mechanically determined OCC vibration pattern affects outer hair cell’s power generation

**Fig 5:**
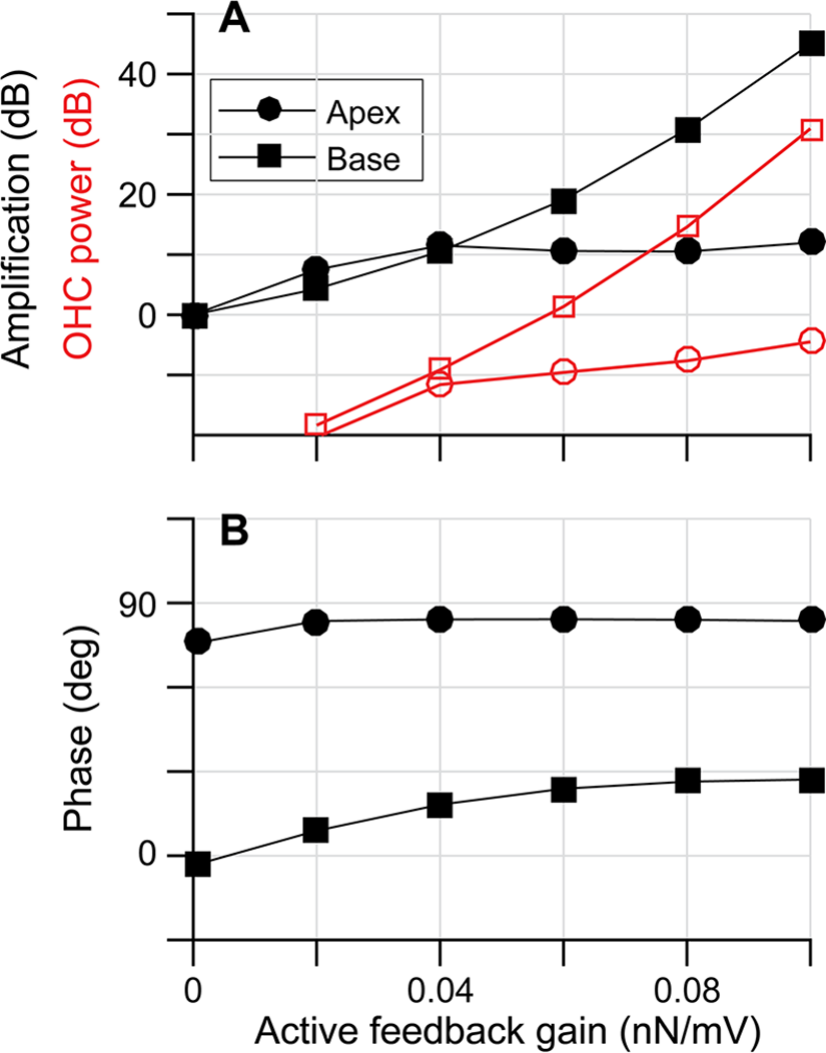
Effect of active gain level on outer hair cell power generation. Different levels of the outer hair cell active force gain (*g*_*OHC*_) were simulated (*g*_*OHC*_ ranges between 0 and 0.1 nN/mV). To represent the two locations, the stapes was stimulated at 15, and 0.7 kHz with 1 nm/ms amplitude. (**A**) Amplification of the BM displacement (black curves). Power generated by an individual hair cell at the peak-responding location versus active feedback gain (red curves). The unit used for power is dB re. 1 fW. (**B**) The phase of the outer hair cell length change rate (*v*_*OHC*_) with respect to its active force amplitude (*f*_*OHC*_) versus active feedback gain.

In Figs 1 and 2, it was shown that the TM and BM vibrate in phase when passive, but the TM leads the BM displacement by 15 to 60 degrees when active. A plausible theory is that the active feedback of the outer hair cells modulates the OCC mechanics to facilitate outer hair cell power generation. We examined whether the outer hair cell active feedback also modulates the phase between *f*_*OHC*_ and *v*_*OHC*_. Different levels of amplification were simulated using different values for the active gain *g*_*OHC*_ (between 0 and 0.1 nN/mV, constant through the cochlear length). The amplification level and the phase between *f*_*OHC*_ and *v*_*OHC*_ were analyzed (Fig 5). As expected, the power generated by the outer hair cell, and the level of amplification increases as *g*_*OHC*_ increases (Fig 5A).

The phase between *f*_*OHC*_ and *v*_*OHC*_ is minimally affected by the level of outer hair cell active feedback (Fig 5B). For high values of *g*_*OHC*_, the basal location is amplified more than the apical location. This location-dependent amplification is consistent with the phase relationships between *f*_*OHC*_ and *v*_*OHC*_: *f*_*OHC*_ is approximately in phase with *v*_*OHC*_ in the basal location, but *f*_*OHC*_ is roughly in phase with the outer hair cell displacement in the apex (panel B), regardless of amplification level. According to this result, the active feedback does not ‘correct’ the phase relationship toward more favorable amplification. That is, the *f*_*OHC*_-*v*_*OHC*_ phase becomes less favorable for amplification as the active gain increases.

**Fig 6:**
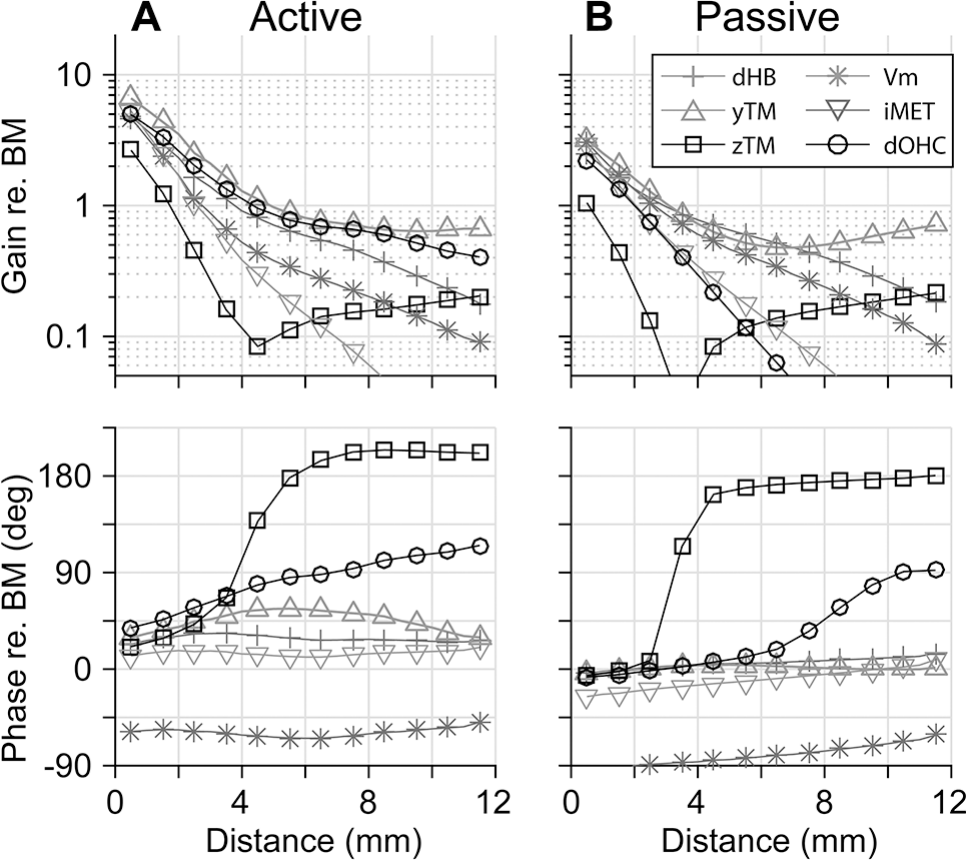
OCC transfer functions relevant to the location-dependent phase relationship. Each data point represents the gain or phase of OCC responses with respect to the BM displacement that were computed for the best frequency at the respective location. *dHB*: hair bundle displacement. *zTM*: TM displacement along the radial (*z)* axis. *yTM*: TM displacement along the transverse (*y*)axis. *Vm*: outer hair cell receptor potential. *iMET*: mechano-transduction current. *dOHC*: outer hair cell length change. All gains are in nm/nm, but mV/nm for *Vm*, and nA/nm for *iMET*. (**A**) Active responses. (**B**) Passive responses.

We investigated which aspect of OCC micro-mechanics is responsible for the location-dependent phase relationship. The amplitudes and phases of six variables with respect to the BM displacement are shown in Fig 6. They are the transverse (*y*) and radial (*z*) displacement of the TM, the mechano-transduction current and receptor potential of the outer hair cells, and the stereocilia and somatic displacement of the outer hair cells. These responses were obtained at various distances from the base, for each location’s best-responding frequency. The following observations were made: First, the gain of most variables decreases toward the apex (top panels of Fig 6). Second, the phases of most variables are approximately flat over the distance (bottom panels of Fig 6). Third, these gain and phase trends are similar with and without outer hair cell feedback (*i*.*e*., the plots of the left and the right columns are similar). Many of the mechanical responses were approximately in phase with the BM vibrations (within ±10 degrees) when the mechanics were passive (gray curves in Fig 6B bottom panel), and were up to 60 degrees out of phase when active (gray curves in Fig 6A bottom panel).

There are two variables that deviate from the general trends. The gain of the TM radial displacement (*z*_*TM*_, curves with □ markers) varies non-monotonically with distance from the base. The gain of *z*_*TM*_ has a minimum value near *x* = 4 mm, and its phase relative to the BM shifts 180 degrees near this location. Although the 180 degree-shift is reminiscent of a resonator, it is not due to resonance. For example, a change of TM stiffness or mass does not change the characteristic location of the phase shift. An explanation for this phase shift is given in the next section.

The phase of the outer hair cell somatic displacement (*d*_*OHC*_, curves with ° markers) increases toward the apex by about 90 degrees with or without outer hair cell feedback. Considering that the phase of the receptor potential (*V*_*m*_, curves with * markers) remains near -60 degrees over the distance when active, the *d*_*OHC*_ phase is primarily responsible for the location-dependent phase difference between *f*_*OHC*_ and *v*_*OHC*_ in Fig 5, since *v*_*OHC*_ *= jωd*_*OHC*_. Note that the trend of the *d*_*OHC*_ phase with distance is not affected by the active feedback of the outer hair cells. To conclude, the extent of the outer hair cell power generation is passive mechanically regulated. The vibration pattern of the OCC represented by the phase of *d*_*OHC*_ is more favorable for amplification in basal locations.

### The direction of TM radial motion depends on geometry

**Fig 7:**
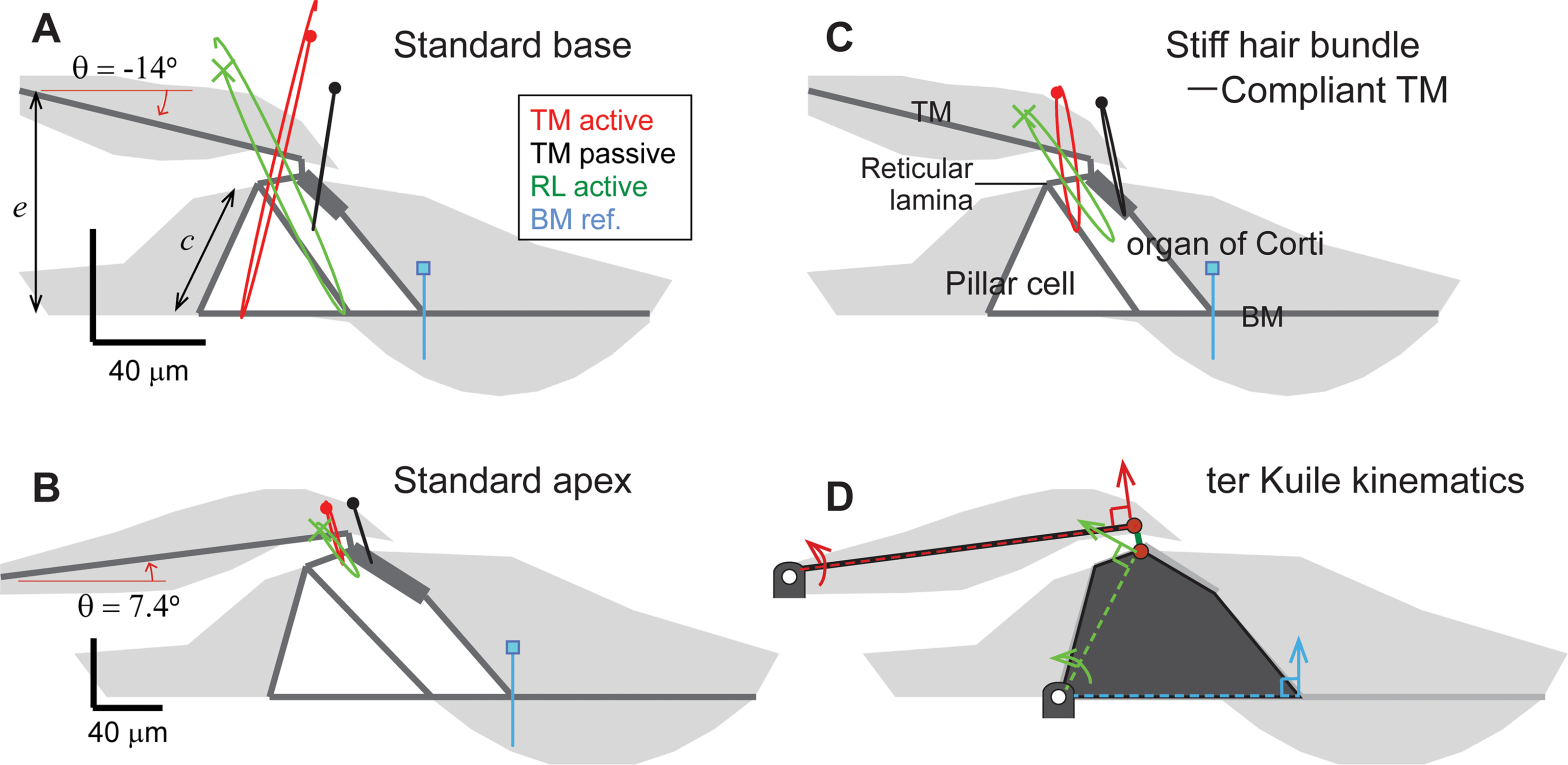
TM radial motion depends on geometrical orientation. Motion trajectories of the TM (red curves when active, black curves when passive), and the reticular lamina (green curves, only active case shown) are referenced to the BM motion (blue curves). The dots indicate the trajectory position at the moment when the BM displacement is maximal toward the scala media. The radial motion of the TM depends on the attachment angle (θ). (**A**) Response at x = 2 mm vibrating at 18.6 (active) and 13.8 kHz (passive). (**B**) Response at x = 10 mm vibrating at 0.81 (active) and 0.64 kHz (passive). (**C**) Stiff hair bundle can reverse the TM radial motion pattern. (**D**) Rigid body kinematics of ter Kuile.

In the present model, the point of attachment between the TM and the spiral limbus is above the reticular lamina in the base (indicated by the dimension *e*, Fig 7A), but it is below the reticular lamina in the apex (Fig 7B). This variation of the TM attachment geometry at different locations is modeled after available anatomical data of the gerbil cochlea [34, 35]. For example, the length of an inner pillar cell is greater than the height of the TM attachment (*c* > *e*) in the apex, but the opposite is true in the base (*c* < *e*) [35].

The variation of the *z*_*TM*_ phase with respect to the BM displacement is determined by geometry, specifically by the attachment angle of the TM (θ in Fig 7). The TM motion trajectories are shown in Fig 7—the red and black curves for active and passive simulations, respectively. The blue curve normal to the BM is the BM trajectory to which the TM trajectory is referenced. For example, the TM to BM amplitude ratio is greater in the basal location than in the apical location. In the basal location, the TM to BM amplitude ratio is greater when active than when passive. The opposite is true in the apical location. The BM vibrates minimally in the radial direction. Roughly speaking, the TM rotates about its attachment point. Because the TM is subject to axial deformation in addition to bending (rotational) deformation, the TM motion trajectory is not exactly normal to the TM. When the OCC kinematics is dominated by the bending deformation, the sign of the attachment angle (θ) determines the direction of *z*_*TM*_. The Meaud-Grosh model is consistent with this interpretation in that it incorporates the bending and the axial motions of the TM explicitly [20, 36]. Further investigation is necessary to learn the functional implications of the bending and axial motion of the TM.

In Fig 7A and 7B, the TM radial displacements are in the positive and negative directions, respectively, for the peak BM upward displacement. In agreement with experimental observations, an angle difference as small as 10 degrees can result in this approximately 180-degree phase reversal of *z*_*TM*_. Although this TM geometry affects the *nominal direction* of the TM radial motion (*z*_*TM*_), we do not find a functional consequence of the geometry, *i*.*e*., the *z*_*TM*_ phase is not correlated with parameters related to cochlear amplification such as the phase of *d*_*OHC*_.

### Parameters that affect the amplification—Sensitivity analysis

**Fig 8:**
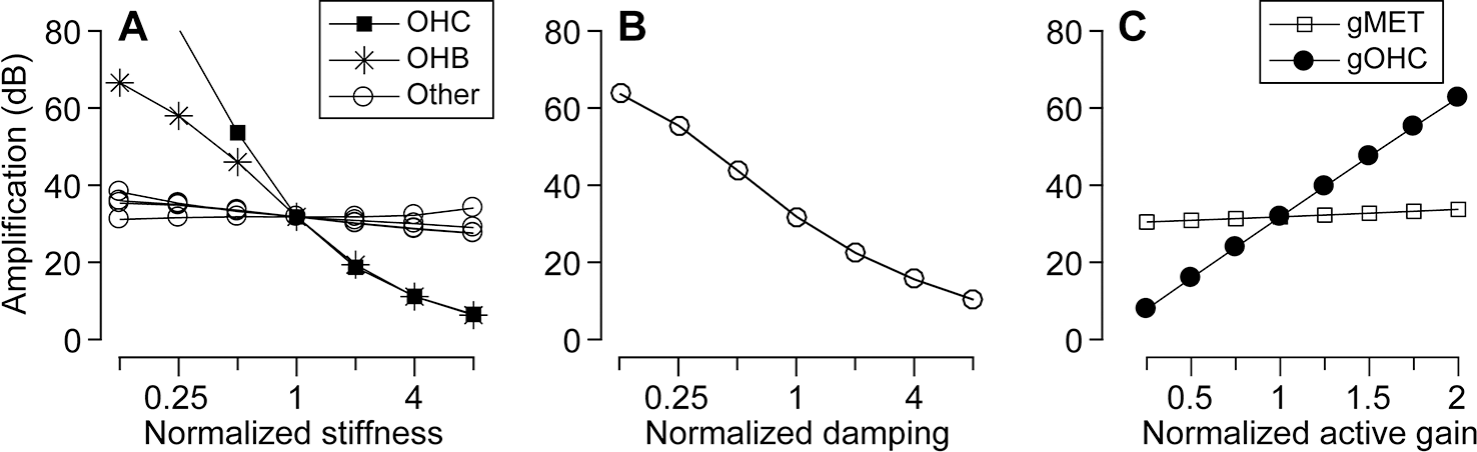
Sensitivity analysis of the model. Each data point represents the amplification factor near *x* = 6 mm with only one model parameter changed from its standard value. (**A**) Effect of component stiffness on amplification. The OCC structures include the outer hair cell body (OHC), outer hair cell stereocilia (OHB), and other structures (Other: the Deiters cell, Deiters cell process, pillar cell, and reticular lamina). Tested values of Young’s modulus of each component range from 1/8 to 8 times the standard value. (**B**) Effect of damping on amplification. (**C**) Effect of the active feedback gains on amplification. Note that the x-axis has a log-scale in (A) and (B), but a linear-scale in (C). The normalized stiffness, damping, and active gain are defined as the altered value divided by the standard value used in the model. Standard values for stiffness and active gain are given in Table 1. Standard values for damping are discussed in the Methods section.

Our model is complex with many independent parameters. Different parameter sets can result in similar functional characteristics, including the level of cochlear amplification. To identify parameters critical for cochlear amplification, a series of sensitivity analyses were performed (Fig 8). With only one model parameter altered from its standard value, amplification factors were obtained. Active and passive responses to a 4 kHz pure tone were used to define the amplification factor. For 4 kHz stimulation, the traveling waves peak in the middle of the model (~6 mm from the base).

The elastic properties of the outer hair cell’s body and hair bundle affect the amplification more prominently than other supporting structures (Fig 8A). As expected, as the level of damping increases the amplification decreases (Fig 8B). Note that this study uses inviscid fluids. Any viscous dissipation in the fluids or in the OCC is approximated with the Rayleigh damping term. The active gain of the somatic motility (*g*_*OHC*_) has a strong effect on the amplification, but the active gain of stereocilia motility (*g*_*MET*_) has negligible effect on the amplification (Fig 8C). This suggests that, in the present model, the somatic motility is the primary active component for amplification.

This series of sensitivity analyses reveals that there exist different sets of parameters that result in the same amplification level. For example, the same level of amplification can be achieved by reducing both OCC damping and active gain, or by decreasing outer hair cell stiffness while increasing OCC damping. The outer hair cell stiffness, the damping imposed on the BM, and the active gain of the outer hair cells are the three most sensitive parameters in our model. As the model parameter increases by a factor of two near the standard value, the amplification level changes approximately by -20 dB, -10 dB and +30 dB, for the outer hair cell stiffness, OCC damping and outer hair cell active force gain, respectively.

Consistent with a previous study (Meaud, Grosh, 2011) and our previous report (Liu et al., 2015), the active hair bundle force minimally affects the OCC mechanics. The somatic force is one to two orders of magnitude greater than the hair bundle force according to available physiological data (Nam, Fettiplace, 2012). For a 1 nm BM vibration amplitude, the *f*_*OHC*_ amplitude ranged between 172 and 11 pN and the *f*_*MET*_ amplitude ranged between 13 and 0.17 pN, over the range of *x* between 2 and 10 mm. The minimal contribution of *f*_*MET*_ to amplification may be ascribed to its small force magnitude. However, even when the active force gain of *f*_*MET*_ (*f*_*MET*,*max*_ in Table 1) was increased by a factor of 10, the contribution of hair bundle forces to amplification remained minimal. This suggests that the somatic force is more favorably situated to deliver power for cochlear amplification. Although we did not observe a significant effect of *f*_*MET*_ on amplification, conditions may exist when the hair bundle force can modulate the OCC mechanics more effectively (*e*.*g*., Ó Maoiléidigh, Hudspeth, 2013).

**Table 1:** Model parameters.

Component	Parameters	x = 2 mm	x = 10 mm	Unit
Basilar membrane	Width (arcuate, pectinate)	53, 107	93, 187	μm
	Thickness (arcuate, pectinate)	0.6, 3	0.14, 0.7	μm
	YM (Radial, Longitudinal)	1000, 0.4	1000, 0.1	MPa
Outer hair cell	Diameter, Length	9, 20	9, 50	μm
	YM	0.045	0.045	MPa
Outer hair cell Hair bundle	Height, Width	2, 8	6, 8	μm
	Stiffness	40	4.5	mN/m
Pillar cell	Diameter	8	4	μm
	YM	10	10	MPa
Deiters cell	Diameter (Body, Phalange)	10, 1.5	10, 1	μm
	YM (Body, Phalange)	0.5, 3	0.5, 3	MPa
Reticular Lamina	Thickness (Pillar cell, OHC)	5, 2	5, 1	μm
	YM (Radial, Longitudinal)	10, 0.2	2, 0.05	MPa
Tectorial Membrane	Width (body, root)	53, 27	140, 70	μm
	Thickness (body, root)	30, 20	50, 25	μm
	Radial YM (body, root)	0.2, 0.8	0.01,0.04	MPa
	Longitudinal YM	0.002	0.002	MPa
Scala	Endocochlear potential, *E*_*e*_	90	90	mV
Stereocilia	Max conductance, *G*_*s*,max_	90	27	nS
	Capacitance, *C*_*s*_	2.6	9	pF
	Resting open prob., *p*_*open*,0_	0.4	0.4	
	Max reactive force, *f*_*MET*,*max*_	100	12	pN
Outer hair cell membrane	Equilibrium potential, *E*_*K*_	75	75	mV
	Resting potential, *v*_*m*,0_	-53	-37	mV
	Conductance, *G*_*m*_	230	39	nS
	Capacitance, *C*_*m*_	4.3	15	pF
	Active gain, *g*_*OHC*_	0.1	0.1	nN/mV

### Mechanical conditions to reverse the location-dependent amplification trend

In theory, the trend of greater amplification in higher frequency locations can be reversed by adjusting model parameters. To determine which parameters have the greatest effect on the location-dependent amplification, we attempted to reverse the location-dependent amplification trend. Although damping or active gain can also affect the trend, we could not find a set of values that completely reverses the amplification trend by adjusting only these parameters. In contrast, the location-dependent amplification trend is readily modulated by adjusting the relative stiffness of the OCC felt by individual outer hair cells.

**Fig 9:**
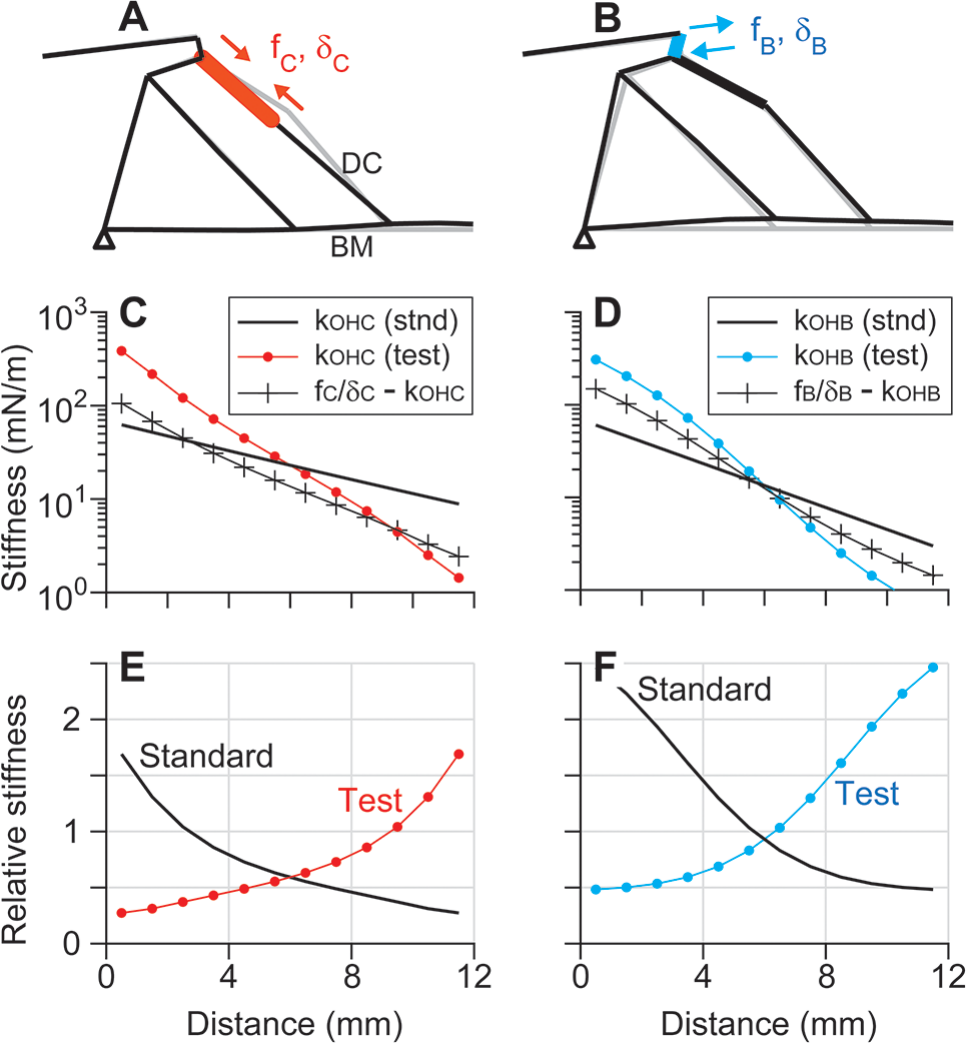
Local stiffness of the OCC. (**A**) Stiffness of the OCC opposing outer hair cell elongation. When a coupled force *f*_C_ is applied at the ends of the outer hair cell, the stiffness felt by the outer hair cell can be calculated from the resultant displacement *δ*_C_. Faint gray lines indicate the non-deformed configuration. (**B**) Stiffness of the OCC opposing hair bundle deflection. (**C**) Standard (thick curve) and test case (curve with ● symbols) values of *k*_OHC_. The OCC stiffness felt by an outer hair cell (curve with + symbols) is also shown. In the test case, the axial stiffness of the outer hair cells was adjusted to obtain the opposite longitudinal trend of *r*_OHC_. (**D**) Standard (thick curve) and test case (curve with ● symbols) values of *k*_OHB_. (**E**) Standard and test case values of *r*_*OHC*_ = (*f*_*C*_*/δ*_*C*_—*k*_*OHC*_) /*k*_*OHC*_. (**F**) Standard and test case values of *r*_*OHB*_ = (*f*_*B*_*/δ*_*B*_*—k*_*OHB*_)/*k*_*OHB*_.

The stiffness of the OCC felt by each outer hair cell has consequences for cochlear amplification [18, 36]. It is the relative stiffness of the OCC as compared to the outer hair cell stiffness that is relevant to power generation by the outer hair cells [37, 38]. To compute the relative OCC stiffness, a set of equal-and-opposite forces was applied to the outer hair cell body (*f*_*C*_) or the stereocilia (*f*_*B*_), and the corresponding static displacements (*δ*_*C*_ or *δ*_*B*_) were obtained (Fig 9A and 9B). The relative OCC stiffness felt by an outer hair cell is defined as *r*_*OHC*_ = (*f*_*C*_*/δ*_*C*_*—k*_*OHC*_)/*k*_*OHC*_, where *k*_*OHC*_ and *f*_*C*_*/δ*_*C*_*—k*_*OHC*_ are the stiffness of the outer hair cell body and the stiffness of the OCC felt by the outer hair cell, respectively. Likewise, the relative OCC stiffness felt by the outer hair cell bundle is defined as *r*_*OHB*_ = (*f*_*B*_*/δ*_*B*_*—k*_*OHB*_)/*k*_*OHB*_, where *k*_*OHB*_ and *f*_*B*_*/δ*_*B*_*—k*_*OHB*_ are the stiffness of the outer hair cell bundle and the stiffness of the OCC felt by the outer hair cell hair bundle, respectively. We introduce the parameters *r*_*OHC*_ and *r*_*OHB*_ for two reasons. First, the stiffness of outer hair cell or its stereocilia bundle has a much greater effect on cochlear amplification than other OCC structures (Fig 8). Second, according to the theory of impedance matching [37, 38], it is the impedance ratio between an actuator and the actuated system that determines the efficiency of power transmission.

We hypothesized that the trend of greater amplification toward the base is a consequence of *r*_*OHC*_ or *r*_*OHB*_ monotonically decreasing toward the apex. To test this hypothesis, while all other model parameters remained the same, either *k*_*OHC*_ or *k*_*OHB*_ was adjusted so that the longitudinal trend of *r*_*OHC*_ or *r*_*OHB*_ was reflected with respect to the center at *x* = 6 mm (curves with dot symbols, Fig 9C and 9F). *k*_*OHB*_ or *k*_*OHC*_ was adjusted instead of other OCC mechanical properties, because a change in the other OCC structures such as the BM or TM stiffness can change the fundamental tonotopy (*i*.*e*., the definition of base and apex will become obscured if the BM stiffness is reversed).

Although we simulated a wide range of outer hair cell stiffness values, it is not because their properties are poorly grounded. The mechanical properties of the outer hair cell and the stereocilia are better understood than other fine structures in the organ of Corti. For example, the hair bundle stiffness has been measured to be ~3 mN/m for 4–5 μm tall outer hair cell hair bundles [39]. Our standard properties (40 and 4.5 mN/m for 2 and 6 μm-tall hair bundles, respectively) are within a reasonable range. The axial stiffness of the outer hair cell body has been measured to be 500 nN per unit strain independent of location [11]. Our standard property is 950 nN per unit strain. We used conservative (greater) *k*_*OHC*_ and *k*_*OHB*_ values than the measured values after considering experimental factors that could influence measured values [e.g., 40]. To reverse the longitudinal trend of *r*_*OHC*_ or *r*_*OHB*_, the ratio between *k*_*OHB*_ values at *x* = 2 and 10 mm is increased from 9 to 140 or the *k*_*OHC*_ ratio is increased from 2.4 to 50. These large variations in stiffness seem unlikely, but are used here as a simple way to illustrate the effect of *r*_*OHC*_ and *r*_*OHB*_.

**Fig 10:**
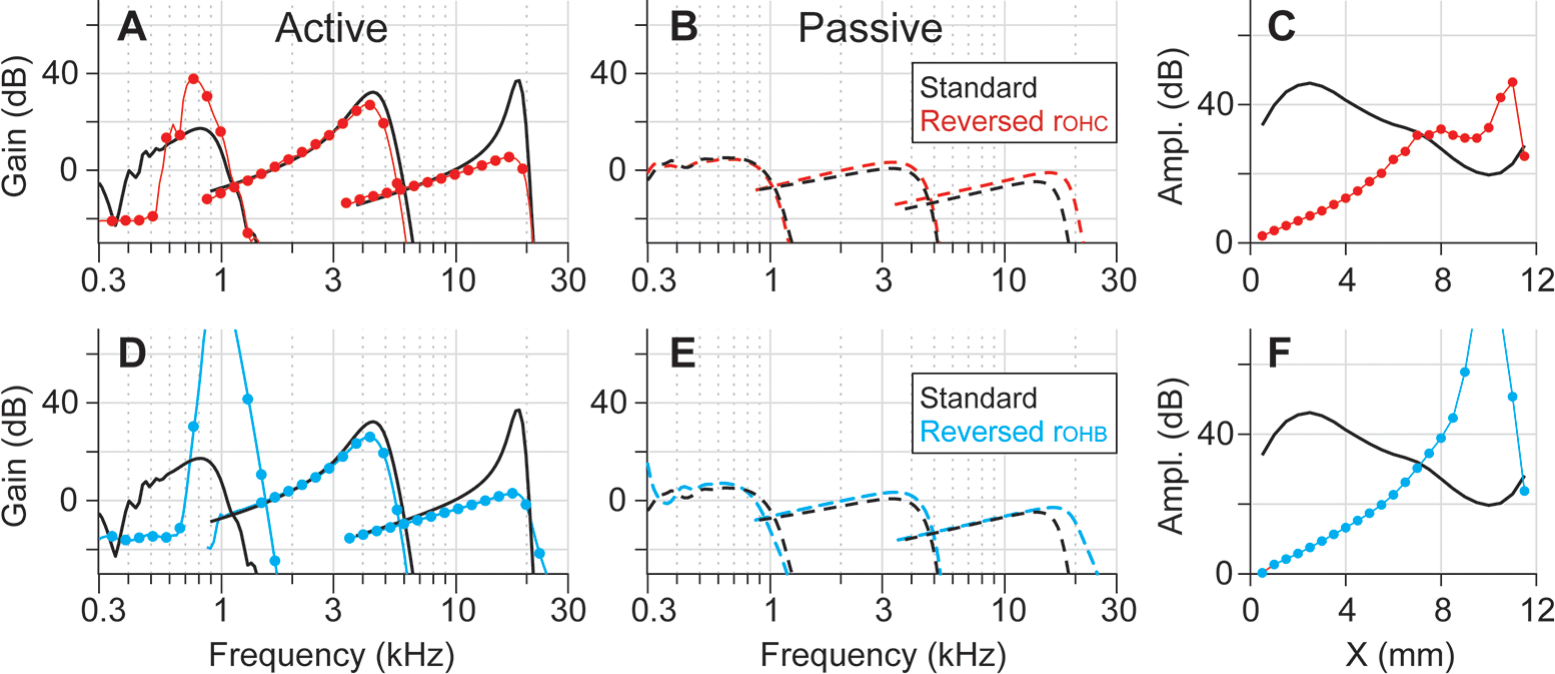
Two mechanical parameters affecting the location-dependent amplification. The black curves are standard responses and the colored curves are from the ‘test’ models with reversed r_OHC_ (**A**-**C**) or r_OHB_ (**D**-**F**) in Fig 9. (**A**) BM displacement gain versus frequency when active. Three pairs of curves are the responses at x = 10, 6, and 2 mm, respectively. With the reversed r_OHC_ model, the apex is amplified more. (**B**) When passive, the reversed r_OHC_ hardly affects the response. (**C**) Amplification levels along the cochlear length. (**D**-**F**) The responses with the reversed r_OHB_ model.

Reversing the longitudinal profile of *r*_*OHC*_ or *r*_*OHB*_ results in a reversed amplification trend along the cochlear length (Fig 10). As a result of this adjustment, the active tuning curves at three locations show less amplification and blunt tuning in the base, greater amplification and sharp tuning in the apex (Fig 10 A and 10D). In contrast to active responses, passive responses were affected minimally by the reversal of *r*_*OHC*_ or *r*_*OHB*_ (Fig 10B and 10E). The amplification level versus location shows that the location-dependent amplification trend is reversed with the adjusted *r*_*OHC*_ or *r*_*OHB*_ (Fig 10C and 10F).

### How *r*_*OHC*_ or *r*_*OHB*_ affects cochlear amplification

**Fig 11:**
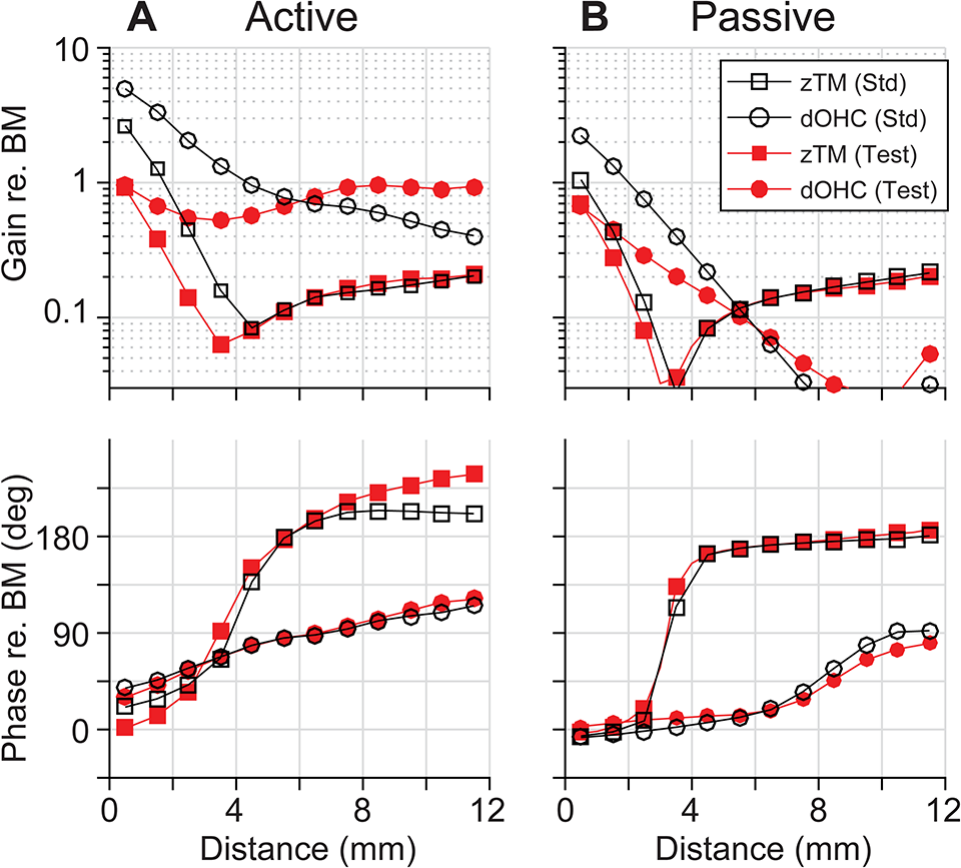
OCC transfer functions—Reversed rOHC. Transfer functions of the TM radial response (zTM) and outer hair cell deformation (dOHC) with the standard parameter set (Std) and reversed r_OHC_ set (Test) when (**A**) active, and when (**B**) passive.

**Fig 12:**
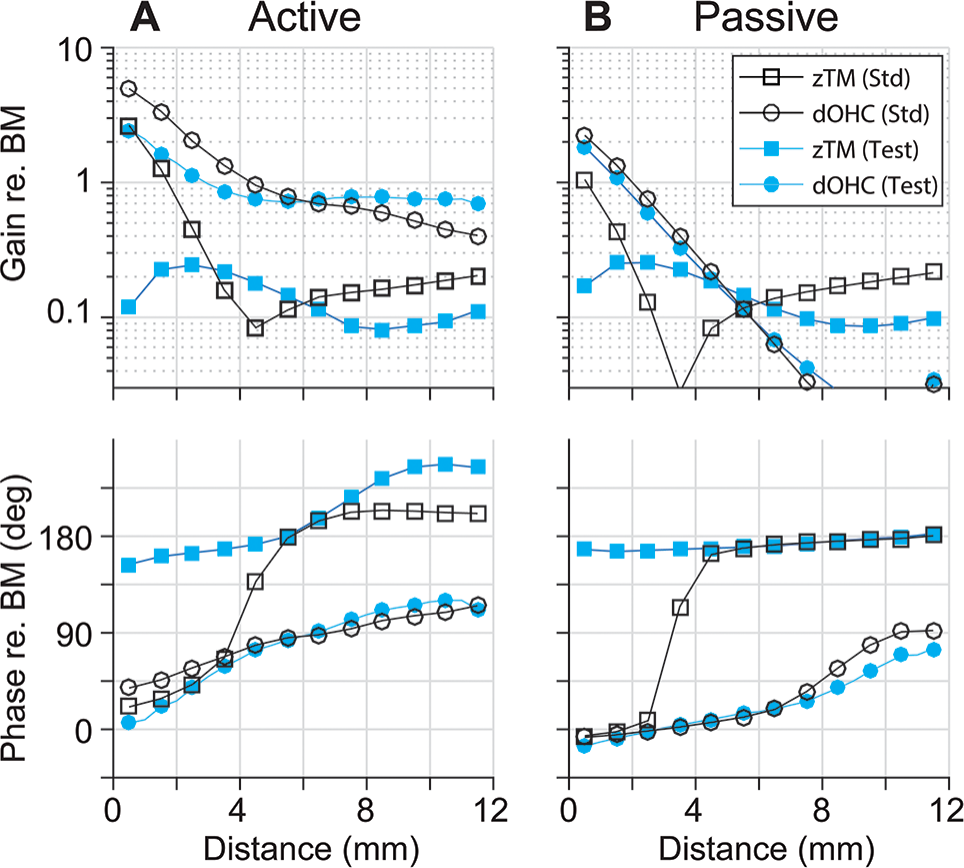
OCC transfer functions—Reversed rOHB. Transfer functions of the TM radial response (zTM) and outer hair cell deformation (dOHC) with the standard parameter set (Std) and reversed r_OHB_ set (Test) when (**A**) active, and when (**B**) passive.

The OCC transfer functions of the test cases with a reversed longitudinal pattern of *r*_*OHC*_ or *r*_*OHB*_ reveal that the two parameters modulate cochlear amplification differently. Figs 11 and 12 present how reversed *r*_*OHC*_ and *r*_*OHB*_ affect the OCC transfer functions. Reversing the spatial pattern of *r*_*OHC*_ affected the outer hair cell length change (the curves with filled circles, top panels of Fig 11), but barely affected the phase relationship. Reversing the spatial pattern of *r*_*OHB*_ affected both the amplitude and phase of the radial TM motion with respect to the BM motion (the curves with ■, Fig 12). The characteristic phase shift of *z*_*TM*_ near *x* = 4 mm disappears because the TM no longer behaves like a rigid bar hinged at the attachment point when the hair bundle stiffness becomes comparable to or greater than the TM axial stiffness (Fig 7C).

**Fig 13:**
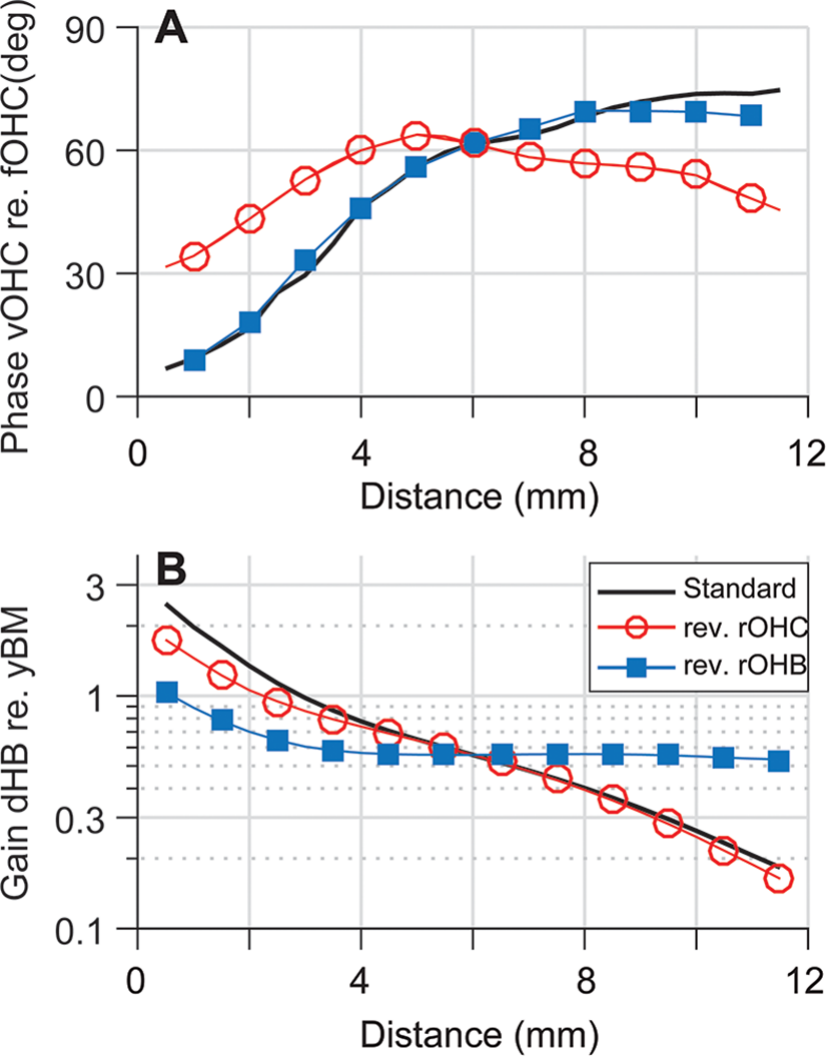
How *r*_*OHC*_ and *r*_*OHB*_ affect cochlear amplification. (**A**) A change in *r*_*OHC*_ results in a change in the *v*_*OHC*_-*f*_*OHC*_ phase relationship (the curve with ○). The plots indicate the *v*_*OHC*_-*f*_*OHC*_ phase difference for the best-responding frequency as a function of location. However, changes in *r*_*OHB*_ minimally affect the *v*_*OHC*_-*f*_*OHC*_ phase relationship (the curve with ■). An outer hair cell can generate the greatest power when *v*_*OHC*_ and *f*_*OHC*_ are in phase. (**B**) A change in *r*_*OHB*_ results in a change in the hair bundle displacement gain. When the longitudinal pattern of *r*_*OHB*_ is reversed, the *d*_*HB*_ gain decreases in the base, but increases in the apex (the curve with ■). However, changes in *r*_*OHC*_ minimally affect the d_*HB*_ gain.

Despite similar outcomes, the mechanisms by which *r*_*OHC*_ and *r*_*OHB*_ affect cochlear amplification are different. Fig 13 summarizes the differences. The change of *r*_*OHC*_ affects the amplification in two ways. First, as the stiffness of the outer hair cell body increases, the effective active force that is used to deform the OCC other than the cell itself decreases. Second, because the outer hair cells act as an elastic coupler between the TM and the BM, the modulation of their stiffness affects the vibration pattern. When the longitudinal dependence of *r*_*OHC*_ is reversed, the phase between *v*_*OHC*_ and *f*_*OHC*_ becomes more favorable for apical power generation as compared to the standard case (the curve with ○, Fig 13A). Unlike the case of reversed *r*_*OHC*_, the reversed *r*_*OHB*_ hardly affected the *v*_*OHC*_-*f*_*OHC*_ phase relationship (the curve with ■, Fig 13A). The change of *r*_*OHB*_ affected the amplification by changing the mechanical gain of the hair bundle displacement (Fig 13B). For example, as a result of decreased *k*_*HB*_ (or increased *r*_*OHB*_), the hair bundle displacement gain (*d*_*HB*_/*y*_*BM*_) was increased from 0.25 to 0.55 at *x* = 10 mm. Because d_*HB*_ is a part of the loop determining the active feedback gain, doubling the hair bundle gain (*d*_*HB*_/*y*_*BM*_) is comparable to doubling the active gain (*g*_*OHC*_). According to Fig 8C, doubling *g*_*OHC*_ resulted in an approximately 40 dB increase in amplification. Unlike the case of reversed *r*_*OHB*_, reversing *r*_*OHC*_ hardly affects the hair bundle displacement gain.

## Discussion

We identified two mechanical conditions that can explain the location-dependent amplification of the cochlea. They are the phase of outer hair cell force with respect to its rate of elongation (Fig 4), and the relative OCC stiffness felt by individual outer hair cells (Fig 10). The phase relationship of the outer hair cell’s motility is determined by passive mechanics: its longitudinal trend is hardly affected by the active feedback of the outer hair cells (Figs 5 and 6). Three model parameters affect the amplification-level most strongly—the outer hair cell’s stiffness, the damping, and the somatic motility (Fig 8). The amplification trend along the cochlear length could be reversed by adjusting the outer hair cell stiffness (Fig 10). In the following, we discuss our finding in the context of other recent studies.

### The origins of location-dependent amplification

Because location-dependent amplification is a well-known characteristic of cochlear physiology, theoretical studies have reproduced this characteristic for validation. However, the origin of location-dependent amplification is quite different from theory to theory, revealing the lack of agreement for the mechanism of cochlear amplification. There are three major aspects to consider: 1) the active force gain of the outer hair cell; 2) the limiting speed of outer hair cell force generation, often referred to as the RC time constant issue; and 3) the timing (phase) of active force application.

Some theoretical studies assumed that the force gain of cochlear actuators varies with location. For example, Mammano and Nobili [5] made two assumptions—the outer hair cell force cancels the damping of BM vibrations, and its amplitude is proportional to the BM stiffness (their Eq (10)). Lu *et al*. [6] used a gain that exponentially varies over the cochlear length to represent the outer hair cell force (parameter *k*_*f*_ in their Table 1). The location-dependent amplification of these studies may represent a graded capacity of the feedback force with phase-locked force application. Although our study used a constant outer hair cell electro-mechanical gain (*g*_*OHC*_ of 0.1 nN/mV), when referenced to the BM displacement like the previous studies, the mechanical and electrical gain of the present model varies with location (Fig 6). In that sense, our study is not inconsistent with the previous studies. Instead, our study divides the active gain into two components: the OCC mechanical gain, and the electro-mechanical gain of the outer hair cells. In this study, we demonstrated that the OCC mechanical gain is location-dependent, and that location-dependent gain affects cochlear amplification.

Recent theoretical studies tend to use an outer hair cell electro-mechanical gain that is independent of location. The Meaud-Grosh model [8, 36], and the Liu-Neely model [7, 41] did not assume increased electromotility of the outer hair cells toward the basal locations. The Liu-Neely model achieves amplification over the physiological frequency range by incorporating high-pass filtering of the mechano-transduction current that compensates for the cell membrane’s low-pass filter. Meaud and Grosh [8] incorporated higher outer hair cell RC filter frequencies (similar to the present study). They used greater mechano-transduction conductance toward high frequency locations, which effectively increased the active feedback gain of the outer hair cell. To summarize, the recent models that include electro-physiological details of the outer hair cells considered either mechano-transduction as a high-pass filter [7, 41] or a location-dependent amplitude modulator [8, 36]. Although the present study focuses on the effect of passive mechanics, it is still worthwhile to further explore this possible role of mechano-transduction.

### Present simulation results in the context of recent experimental observations

The experimental study by Dong and Olson [16] share a conclusion with our study—the passive mechanics modulate cochlear amplification. Their measurements are valuable in that both electrical and mechanical responses of intact OCC at the site of amplification were presented. Similar to our results (Fig 6), the action of outer hair cells only modestly affects the phase relationship between electrical and mechanical responses. At first glance, Dong and Olson’s results seem incongruent with Chen *et al*.’s measurements [13]: the TM-BM phase relationship was observed to vary with stimulus level. According to our results, those two observations are not necessarily incongruent. For example, the TM vibration phase with respect to the BM varies depending on the outer hair cell feedback (Figs 1 and 2) similar to Chen *et al*.’s observation. The timing of outer hair cell’s force generation (*v*_*OHC*_-*f*_*OHC*_ phase relationship) is passive mechanically determined (Figs 5, 6 and 11) in line with Dong and Olson’s conclusion.

Recent advances in measurements of the OCC micro-mechanics began to provide data that previously unavailable such as tissue vibrations in the apical turn, and relative motions between OCC fine structures. Ren and his colleagues have refined low-coherence heterodyne laser interferometry to measure vibrations of both the top and the bottom surfaces of the OCC in live mouse cochlea [15, 42]. When the cochlea was insensitive the motions of the OCC fine structures were in-phase. In contrast, the motions of OCC structures showed frequency-dependent phase differences in sensitive cochlea. Oghalai and his colleagues used optical coherence tomography to measure the OCC vibrations of live mouse cochlea [14, 43]. Similar to Ren *et al*.’s observation, the OCC structures tended to vibrate more in unison when the cochlea is passive (dead) than when it is active (sensitive). Our results are in qualitative agreement with those measurements in that active responses show more complex vibration patterns than the passive case as seen from the phase differences in Fig 6. For now, however, direct comparison may need caution because of the differences in animal models. Those recent measurements are from the mouse cochlea which has a higher frequency range than our subject (the gerbil cochlea). We chose the gerbil cochlea because its mechanical properties are well-known. For example, for model studies, the BM stiffness is an essential parameter that determines the frequency-location relationship. The BM stiffness at different locations has been available only for the gerbil cochlea [44, 45] until recent measurements for the mouse cochlea [46]. Our simulated results compare well with measurements from the gerbil cochlea by Chen *et al*. (2011). For example, the TM vibrations leads the BM vibrations as the outer hair cells’ feedback becomes more prominent (Figs 1 and 2). The amplitude ratio between the TM and the BM transverse vibrations is greater when active than passive.

### A consequence of the relative OCC stiffness (*r*_*OHC*_ and *r*_*OHB*_) being near unity

**Fig 14:**
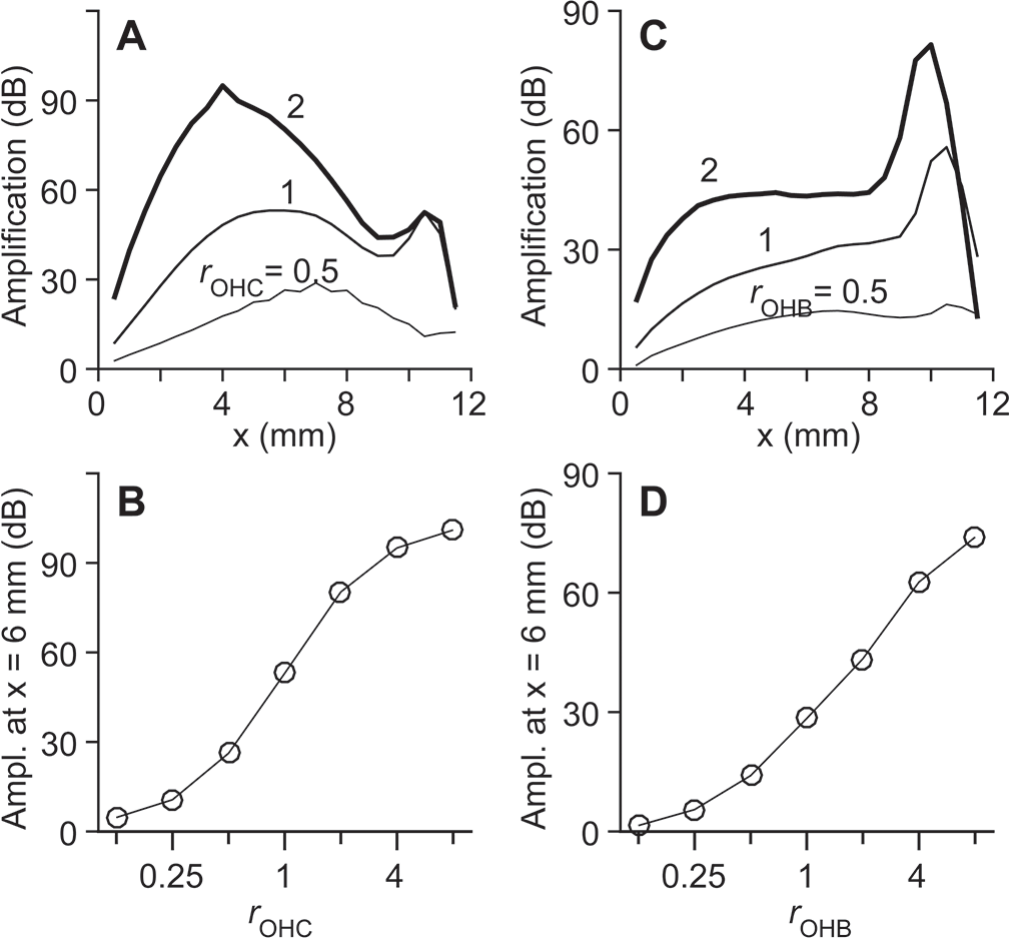
Effect of outer hair cell stiffness on cochlear amplification. The stiffness of the outer hair cells or their stereocilia bundles was adjusted so that *r*_OHC_ or *r*_OHB_ was constant over the cochlear length. (**A**) Different *r*_OHC_ values result in different amplification levels over the cochlear length. (**B**) Amplification level at *x* = 6 mm for different *rOHC* values.(**C** and **D**) Effect of different *r*_OHB_ values on amplification.

There may be a consequence of the relative stiffness *r*_*OHC*_ or *r*_*OHB*_ values being near unity. We investigated how different values of *r*_*OHC*_ or *r*_*OHB*_ affect amplification (Fig 14). To focus on the effect of the magnitudes of these two parameters instead of their longitudinal variations, in this series of simulations, the *r*_*OHC*_ or *r*_*OHB*_ values were set constant along the cochlear length. Although our model is linear (*e*.*g*., the transduction current does not saturate), the amplification level saturates as *r*_*OHC*_ or *r*_*OHB*_ increases by an order of magnitude (or the outer hair cell stiffness is reduced by one order of magnitude). For example, when the outer hair cells are very compliant (*r*_*OHC*_ >>1) or stiff (*r*_*OHC*_ <<1) as compared to their surrounding structure, a small change in outer hair cell stiffness does not affect the level of amplification. There exists an optimal range where changes of *r*_*OHC*_ or *r*_*OHB*_ have the greatest influence on amplification. Our result suggests that, if the active motility of the stereocilia or the cell body modulates its stiffness, those actions will result in level-dependent amplification. If such a level-dependent modulation exists, it is most effective when *r*_*OHC*_ or *r*_*OHB*_ is near unity where the modulation sensitivity (the slope of the curves in Fig 14B and 14D) is greatest. Previous studies foresaw a similar condition (unity *r*_*OHC*_) for efficient power generation by outer hair cells [37, 38]. According to physiological evidence, the stiffness of the outer hair cell’s hair bundle is dependent on the stimulation level [39, 47].

## Methods

### Mechano-electrical model of the cochlea

**Fig 15:**
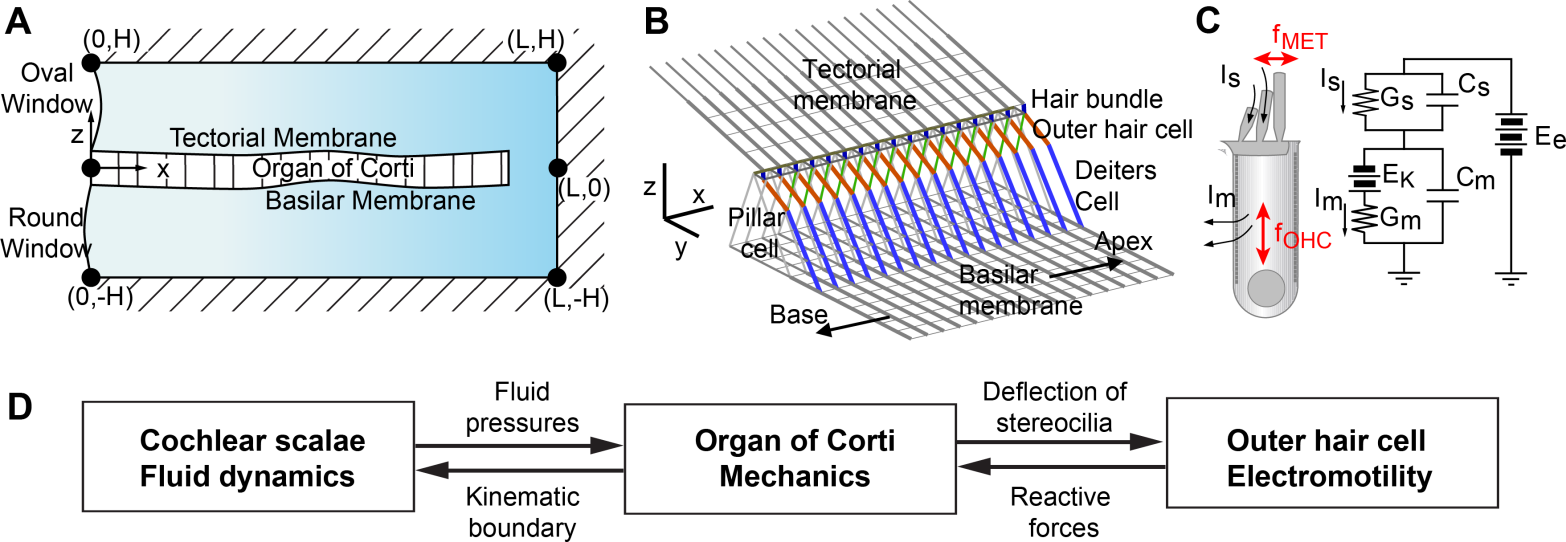
Multiscale model of the cochlea. (**A**) Cochlear fluid dynamics: The cochlear cavity is represented by a fluid-filled rectangular space divided into the top and the bottom fluid compartments separated by the elastic OCC. (**B**) OCC micro-mechanics: The 3-D finite element model of the OCC incorporates realistic geometrical and mechanical characteristics of the gerbil cochlea. The OCC micro-structures repeat with a longitudinal grid size of 10 μm. (**C**) Outer hair cell mechano-transduction and electro-mechanics: Two active forces are incorporated with the outer hair cells—the force originating from mechano-transduction in the hair bundle (*f*_MET_) and the electromotive force of the cell membrane (*f*_OHC_). (**D**) Interactions between the three dynamic systems.

The same cochlear mechano-electrical model as Liu *et al*.’s [18] was used for this study with some adjustments of parameters (Fig 15, Table 1). When possible, physiological parameters were obtained from existing gerbil cochlea data. Three dynamic systems were solved simultaneously—the fluid dynamics of the cochlear scalae, structural mechanics of the OCC, and hair cell electro-physiology.

The cochlear fluid domain is represented by a 2-D rectangular space separated by the OCC into two compartments (Fig 15A). The basal end of the two compartments (*x* = 0) represents the oval window (0 < *z* < *H*) and the round window (-*H* < *z* < 0), where *H* is the compartment height. Throughout this work, the oval window was subjected to a constant velocity amplitude (1 nm/ms, corresponding to ~50 dB SPL in the ear canal [24, 48]) independent of stimulating frequency. The round window is considered to be a pressure-release (zero pressure) boundary. All other external fluid boundaries are considered rigid. At the end of the cochlear coil, an opening called the helicotrema connects the top and the bottom fluid domains. Fluids in the top and the bottom compartments interact separately with the OCC through its top and the bottom surfaces. The bottom interacting surface is represented by the mid-line of the BM. The top interacting surface is represented by the TM edge located over the outer hair cell hair bundles. When the cochlea is passive, because the two interacting surfaces vibrate in phase, the predicted response is similar to models using a single interacting surface.

The structural mechanics of the OCC is solved using a 3-D finite element method. Structurally significant components of the OCC such as the TM, BM, outer hair cells, pillar cells, Deiters’ cells, and reticular lamina are incorporated [27]. Because each fine structure of the OCC has a clearly defined primary axis (the direction with which microtubules, actin fibers or collagen fibers are aligned), beam elements are used to represent these micro-structures. Simplifications of the OCC mechanics include: three rows of the outer hair cells are merged into one; and non-structural supporting cells and inner hair cells are neglected. The fine structures of the OCC reflect their anatomical dimensions, configurations, and mechanical properties that vary along the cochlear length. The BM is hinged along the spiral lamina and clamped along the spiral ligament. The edge of the TM along the spiral limbus is clamped. The apical and the basal extremities of the OCC (*x* = 0 and 12 mm) are clamped. This boundary conditions at *x* = 0 and 12 mm have negligible effect on the results. The OCC is subjected to two different types of stimulating force. One is the hydrodynamic pressures acting on the top and the bottom surfaces of the OCC. The other is the active forces from the outer hair cells.

Viscous damping matrix is defined by multiplying the stiffness matrix by a coefficient (stiffness-proportional Rayleigh damping coefficient). The damping coefficient varies independently with longitudinal position. The coefficient value is chosen to obtain a reasonable tuning-quality factor with the passive model. The Rayleigh damping coefficient was *α*_*C*_ = 0.963exp(0.413*x*) μsec, where the distance *x* is in mm. With this value, the passive cochlea is slightly under-damped (Q_10dB_ 1.1 at the base and 0.9 at the apex). This value is comparable to 2.2–0.23 kN·s/m/m^2^ when considered per unit BM area. The damping coefficients for two components are independently determined: First, the viscous dissipation coefficient for the sub-tectorial space is analytically approximated, assuming Newtonian viscous friction, by *c*_*STS*_ = μ*Lw/h*, where *L*, *w* and *h* are the length, width and height of the space. The dynamic viscosity μ was 0.7 mPa·s. This value was added to the dissipating component of the outer hair cell stereocilia bundles. Second, viscoelastic properties of the outer hair cells were determined based on the literature (13). The assigned value of the outer hair damping coefficient ranges between 0.3 μN·s/m in the base and 0.75 μN·s/m in the apex.

The active mechanical feedback of the outer hair cells is represented by two electro-mechanical components: mechano-transduction of the stereocilia and electro-mechanical motility of the basolateral membrane. The mechano-transduction in the outer hair cell stereocilia is driven by the relative displacement between the TM and the reticular lamina which is equal to the shear displacement of the hair bundle. The transduction channel kinetics are based on previous studies [39, 49]. The product of the gating swing (0.7 nm) and gating spring stiffness (6 mN/m) determines the transduction sensitivity. The electrical circuit of the outer hair cell comprises the conductance of the stereocilia (G_s_) and the lateral membrane (G_m_), the capacitance of the stereocilia (C_s_) and the membrane (C_m_), and collective equilibrium potential (E_K_). The electrical conductance of outer hair cell’s basolateral membrane are based on the literature [50]. The resultant resistor-capacitor (RC) corner frequency of the outer hair cell membrane ranges from 0.2 kHz in the apical end to 15 kHz in the basal end. As this RC corner frequency is lower than the best responding frequency along the cochlear length, the receptor potential lags the mechano-transduction current by about a quarter cycle (cf. the phase difference between iMET and Vm in Figs 1D and 2D and Fig 6), indicating that the outer hair cell impedance is largely capacitive. The outer hair cell membrane generates a force proportional to membrane potential change. A constant electro-mechanical gain of 0.1 nN/mV [11] is used.

Model parameters are obtained primarily from the experimental data of the gerbil cochlea (Table 1). The simulation code was written and implemented in Matlab (Mathworks, Natick, MA). The longitudinal (along the *x*-axis) grid size is 10 μm matching the spacing between the outer hair cells. To match the structural domain grid, the mesh grid size of the fluid domain is also 10 μm. The problem size of the combined system was about 0.23 million degrees-of-freedom. When run on an IBM PC (Intel i7-4790 processor, 16 GB RAM), it takes 3 minutes to assemble matrices and 20 seconds to solve for each stimulating frequency. The simulation code is available through the research webpage of the corresponding author.

### Power flux in the scalae

The power flux in the fluid through a cross-section at the distance *x* averaged over a cycle is

Pflux(x)=0.5w∫−HHRe(pslowvfCT)dz, (1)

where *w* is the width of cochlear scalae (compartment), *v*_*f*_ is the fluid velocity magnitude in the longitudinal direction, the superscript CT indicates the complex conjugate, and *p*_slow_ represents the pressure component caused by the slow traveling waves on the OCC. A positive value of *P*_*flux*_ indicates the flow toward the apex of the cochlea. The same definition was used in a previous study [51]. Little energy from the slow traveling wave reaches the helicotrema, when the wave peaks and decays before reaching the helicotrema. That is, the pressure at the helicotrema *p*(*L*,*0*) approximates the fast wave pressure. This fast wave pressure is subtracted from the total pressure to obtain the slow wave pressure, or *p*_slow_(*x*,*z*) *= p*(*x*,*z*) *− p*(*L*,*0*). *p*_slow_ is equivalent to the fluid pressure of other studies that assume an anti-symmetric pressure. Because the fluid velocity is not explicitly obtained in our inviscid fluid model, *v*_*f*_ is obtained from the pressure field.

$$v_{f}=-\left(1/j\omega \rho \right)\left(\partial p_{slow}/\partial x\right).$$ (2)

### Power provided and dissipated in the cochlea

The present cochlear system has two power sources. The stapes vibrations excite the system. In addition, the outer hair cells supply power to locally amplify the OCC responses. As we assumed that the fluid is inviscid, all the provided power is dissipated within the OCC through the viscous damping represented by the damping matrix **C** in Eq (A3) in S1 Appendix. The dissipated power in the cochlea is calculated from the OCC velocity **v**,

$$P_{loss}=0.5v^{CT}Cv.$$ (3)

The power provided by the stapes is calculated from the pressure and velocity at the stapes (*x* = 0, 0 < *z* < *H*),

Pstapes=0.5wstapes∫0HRe(p(0,z)vfCT(0,z))dz, (4)

where *w*_*stapes*_ is the effective width of stapes in the radial direction assuming the fluid velocity component *v*_*f*_ is uniform in the radial direction. Meanwhile, the power provided by the fluid to the OCC is

Pf2s=0.5∫0LRe(ffluid(x)vCPCT(x))dx, (5)

where *v*_*CP*_ is vector containing the transverse velocities at the fluid-structure interfaces (*i*.*e*., the TM and the BM velocity for the top and the bottom surfaces, respectively). When there are no active forces of the outer hair cells, the power delivered through the stapes is equal to the power provided by the fluid to the structure, or *P*_*stapes*_ *= P*_*f2s*_. The effective width of the stapes (*w*_*stapes*_) is obtained from this equality and used for the active cochlea cases, too. The power provided by the somatic motility of a single outer hair cell is the product of the outer hair cell force (*f*_*OHC*_), and the rate of outer hair cell elongation (*v*_*OHC*_),

POHC=0.5Re(fOHCvOHCCT). (6)

There are 3 outer hair cells per a 10 μm section, and there are total of 1201 sections.

## Supporting information

S1 Appendix Governing equations of coupled fluid-structure-electrical system. (DOCX)
