# Supplementary material for: Two passive mechanical conditions modulate power generation by the outer hair cells
Source: PLoS Comput Biol. 2017 Sep 7;13(9):e1005701. doi: 10.1371/journal.pcbi.1005701 (PMC5604991; doi:10.1371/journal.pcbi.1005701)
Supplement: S1 Appendix — (DOCX) [file pcbi.1005701.s001.docx]

## S1 Appendix. Governing equations of coupled fluid-structure-electrical system

Assuming small amplitude sinusoidal excitation about the resting state of the coupled system, the fluid dynamics, structural mechanics and outer hair cell electro-mechanics are solved simultaneously. The fluid domain is discretized with a 2-D finite difference scheme (uniform mesh size of 10 μm). The structural domain of the OCC is discretized using a 3-D finite element method. By assuming small vibrations of the incompressible and inviscid fluid, the governing equation for the fluid dynamics (the Navier-Stokes equations) is reduced to the Laplace equation for pressure,

$\nabla^{2}p=0$ (A1)

where is the Laplace operator and *p* is the pressure. After discretization of the fluid domain, the equation is expressed in matrix form as

$\mathbf{A}_{pp} \mathbf{p}+\mathbf{A}_{px}\mathbf{x}=\mathbf{b}_{OW}$ (A2)

where **A***_pp_* corresponds to the Laplace operator, **A***_px_***x** and **b***_OW_* are terms that account for the structural boundaries of the OCC, and the kinematic boundary including the oval window, respectively, and **x** is the transverse displacements along the length of the OCC.

When the system is subjected to sinusoidal stimulation with angular frequency of *ω*, the governing equation for the OCC structural dynamics is written in terms of mass (**M**), damping (**C**) and stiffness (**K**) matrices as

$(\mathbf{K}-\omega^{2}\mathbf{M}+j\omega\mathbf{C})\mathbf{x}=\mathbf{f}_{fluid}+\mathbf{f}_{OHC}+\mathbf{f}_{MET}$ (A3)

where $j$ is the unit imaginary number. The three terms on the right hand side of Eq. (3) are the force vectors due to fluid pressure, outer hair cell somatic motility, and stereocilia bundle motility, respectively. The damping matrix **C** was approximated as **C** *= α_C_***K**, where the coefficient varies exponentially along the cochlear length.

After linearization about the resting states, the amplitudes of mechano-transduction current *i*_s_ and membrane potential *v*_m_ of an outer hair cell are proportional to the stereocilia bundle displacement and the transduction current, respectively, or

$i_{S}=\alpha_{MET}x_{s}$, and $v_{m}=\alpha_{OHC}i_{s}$. (A4, A5)

The coefficients of α*_MET_* and α*_OHC_* are evaluated at the resting state, and they are dependent on frequency and location (Liu *et al*., 2015). The active forces of the stereocilia and the membrane are proportional to *i_S_* and *v_m_*, respectively, so

$f_{MET}=g_{MET}i_{s}/i_{s,max}$, and $f_{OHC}=g_{OHC}v_{m}$. (A6, A7)

In the present study, the stereocilia force gain *g_MET_* ranges between 20 pN (apical end) and 100 pN (basal end), and the somatic force gain *g_OHC_* is 0.1 nN/mV independent of location. After introducing a vector of the electrical variables **e** = [**i**_S_, **v**_m_]^T^, Eqs. (4) and (5) can be expressed in matrix form as

$\mathbf{A}_{ex}\mathbf{x}+\mathbf{A}_{ee}\mathbf{e}=0$. (A8)

From Eqs. (S6) and (S7), the active forces in Eq. (S3) can also be expressed in terms of the electrical variable vector **e** as

$\mathbf{f}_{OHC}+\mathbf{f}_{MET}=\mathbf{A}_{xe} \mathbf{e}$. (A9)

The force due to the fluid pressure is obtained from the effective areas of the OCC (**A**_xp_) and the pressure.

$\mathbf{f}_{fluid}=\mathbf{A}_{xp} \mathbf{p}$. (A10)

From Eqs. (S3), (S9) and (S10), the equations of motion become

$\mathbf{A}_{xx}\mathbf{x}=\mathbf{A}_{xp} \mathbf{p}+\mathbf{A}_{xe} \mathbf{e}$, (A11)

where **A***_xx_* = **K** – *ω*^2^**M** + *jω***C**.

Eqs. (S2), (S9) and (S11) represent the fluid dynamics of the cochlear scalae, outer hair cell electro-mechanics, and OCC mechanics, respectively. Those three equations are coupled together and can be written in matrix form.

$\left[\begin{matrix} \mathbf{A}_{pp} & \mathbf{A}_{px} & 0 \\ \text{-}\mathbf{A}_{xp} & \mathbf{A}_{xx} & \text{-}\mathbf{A}_{xe} \\ 0 & \mathbf{A}_{ex} & \mathbf{A}_{ee} \end{matrix}\right]\left[\begin{matrix} \mathbf{p} \\ \mathbf{x} \\ \mathbf{e} \end{matrix}\right]=\left[\begin{matrix} \mathbf{b}_{ow} \\ 0 \\ 0 \end{matrix}\right]$. (A12)
